# Allometries of Maximum Growth Rate versus Body Mass at Maximum Growth Indicate That Non-Avian Dinosaurs Had Growth Rates Typical of Fast Growing Ectothermic Sauropsids

**DOI:** 10.1371/journal.pone.0088834

**Published:** 2014-02-25

**Authors:** Jan Werner, Eva Maria Griebeler

**Affiliations:** Department of Ecology, Zoological Institute, University of Mainz, Mainz, Germany; Raymond M. Alf Museum of Paleontology, United States of America

## Abstract

We tested if growth rates of recent taxa are unequivocally separated between endotherms and ectotherms, and compared these to dinosaurian growth rates. We therefore performed linear regression analyses on the log-transformed maximum growth rate against log-transformed body mass at maximum growth for extant altricial birds, precocial birds, eutherians, marsupials, reptiles, fishes and dinosaurs. Regression models of precocial birds (and fishes) strongly differed from Case’s study (1978), which is often used to compare dinosaurian growth rates to those of extant vertebrates. For all taxonomic groups, the slope of 0.75 expected from the Metabolic Theory of Ecology was statistically supported. To compare growth rates between taxonomic groups we therefore used regressions with this fixed slope and group-specific intercepts. On average, maximum growth rates of ectotherms were about 10 (reptiles) to 20 (fishes) times (in comparison to mammals) or even 45 (reptiles) to 100 (fishes) times (in comparison to birds) lower than in endotherms. While on average all taxa were clearly separated from each other, individual growth rates overlapped between several taxa and even between endotherms and ectotherms. Dinosaurs had growth rates intermediate between similar sized/scaled-up reptiles and mammals, but a much lower rate than scaled-up birds. All dinosaurian growth rates were within the range of extant reptiles and mammals, and were lower than those of birds. Under the assumption that growth rate and metabolic rate are indeed linked, our results suggest two alternative interpretations. Compared to other sauropsids, the growth rates of studied dinosaurs clearly indicate that they had an ectothermic rather than an endothermic metabolic rate. Compared to other vertebrate growth rates, the overall high variability in growth rates of extant groups and the high overlap between individual growth rates of endothermic and ectothermic extant species make it impossible to rule out either of the two thermoregulation strategies for studied dinosaurs.

## Introduction

How fast does an organism grow? This is an important question because individual growth is linked to many life history traits. For example, age at sexual maturity and mortality rate in addition to metabolic rate and reproductive output vary with age and body mass [1]–[6]. The growth of an animal is determined by its energy intake (metabolic rate) and the allocation of metabolic energy to individual growth and other demands, like maintenance and reproduction [7], [8]. Case [9], [10] showed that endotherms have higher maximum growth rates than ectotherms, and suggested that endothermy (in conjunction with high metabolic rates and parental care) accounts for this observation. Since Case’s [9], [10] papers, many studies have been released dealing with the growth rates of animals and an ever increasing amount of data on growth in extant vertebrates and non-avian dinosaurs is available today [11]–[13]. However, to the best of our knowledge, a comparison between different taxonomic groups, similar to Case [10], was never done again. Only growth rates of single taxonomic groups (mainly mammals and birds) were studied and compared to Case’s regression models [10], [12], [14]. However, different mathematical methods were used to calculate growth rates of individuals; for this reason, comparisons might be difficult between studies. Even within his study, Case [9] used different methods to determine maximum growth rates of individuals from the studied taxonomic groups. Today, more objective methods (in terms of mathematics) to estimate growth rates are available.

Case [9], [10] used maximum absolute growth rate (AGR) to assess maximum growth and linked that to (asymptotic) body mass. This is problematic because maximum growth can occur at different body mass (BM) proportions (e.g. at 30% and at 50% of asymptotic body mass) for species with similar asymptotic body masses. A higher AGR of one species compared to another can result only because the maximum growth rate occurs at a higher body mass proportion, even if the relative growth rate (RGR) of this species is the same as or lower than for the other species (RGR is maximum AGR through the BM where the maximum AGR occurs).

Today, a proper method for estimating growth rates is to fit non-linear growth functions to growth data. The most commonly used growth models describing individual growth are the Logistic, Gompertz or von Bertalanffy growth functions [12]–[19]. All three functions are similar in shape (sigmoidal), but the location of the point of inflection differs. The von Bertalanffy function has a point of inflection at approximately 30%, the Gompertz function at around 37% and the Logistic function at 50% of asymptotic body mass. Thus, maximum growth, which is observed at the point of inflection and is expressed in absolute maximum growth rate, is not comparable even between these standard models without an appropriate transformation.

In our study, we tested if Case’s [9] statements that (i) “the evolution of endothermy was a key factor in lifting physiological constrains upon growth rate” and (ii) “the maximum observed growth rates of endotherms (except for some marsupials and anthropoid primates) are at least an order of magnitude greater than the maximum growth rate of any ectotherm” are still valid when analyzing a much larger dataset on growth rates in extant vertebrate taxa. To do this, we established phylogenetic generalized regression equations (PGLS) on maximum growth rate and body mass at maximum growth (BMatMG) for different extant taxonomic groups (altricial birds, precocial birds, eutherians, marsupials, reptiles, fishes; Note: if we use the term reptile(s) in this study we mean extant reptiles exclusive of birds, if we use the term bird(s) we only mean recent bird species, if we use the term dinosaur(s) we mean non-avian dinosaurs excluding recent birds). To minimize the drawback of using different methods for calculating growth rates, we only used growth rate constants of fitted growth models. Additionally, we standardized the growth rate constants of growth models (using AGR instead of the growth rate constant k and regressed AGR to the body mass where maximum growth occurred, instead of using adult body mass or asymptotic body mass as was done by Case [9], [10]; (for an introduction to growth models and their parameters see [15]). First, we calculated PGLS regressions on log-log-transformed data and tested if the slopes of our regressions were consistent with the theoretically assumed value of 0.75 (AGR should scale as metabolic rate with 0.75 [5], [20]). Second, we calculated the normalization constants for each of the different taxonomic groups (see Results in Table 1 and 2) using a slope of 0.75 as a fixed value in our regression model. Additionally, we compared growth rates of the studied extant species with those of several dinosaurs [21]. In the case that dinosaurs had growth rates that are much higher than observed in any (scaled-up) ectothermic species and Case’s statement (i) is valid, then strong evidence would be provided that dinosaurs were already endotherms. The last step taken was the comparison of our regressions with those presented in Case [10].

**Table 1:** Phylogenetic generalized least square linear regression models (PGLS) on maximum absolute growth rate per day (AGR) against body mass at maximum growth (BMatMG, in gram) for different taxonomic groups.

group	N	intercept	95% CI	p-value	slope	95% CI	p-value	lambda	AIC
altrical birds	343	−0.610	[−0.719, −0.501]	<2e-16	0.803	[0.766, 0.840]	<2e-16	0.945	−519.804
precocial birds	164	−0.830	[−1.048, −0.612]	<2e-16	0.746	[0.691, 0.800]	<2e-16	0.860	−175.392
eutherians	299	−1.052	[−1.230, −0.876]	<2e-16	0.710	[0.670, 0.750]	<2e-16	0.815	−62.491
marsupials	21	−1.297	[−1.470, −1.124]	<2e-16	0.784	[0.722, 0.847]	<2e-16	−0.305	−3.263
non-avian dinosaurs	19	−1.946	[−2.458, −1.435]	<2e-16	0.783	[0.696, 0.870]	<2e-16	0.933	−7.751
reptiles	35	−2.143	[−2.651, −1.636]	<2e-16	0.694	[0.556, 0.832]	<2e-16	0.543	58.153
fish	30	−2.800	[−3.259, −2.341]	<2e-16	0.867	[0.789, 0.944]	<2e-16	0.741	13.982

AGR and BMatMG were log 10 transformed before PGLS was conducted (log10 AGR = log10 intercept+log10 BMatMG * slope). Models are ordered by values of intercepts. N = sample size. CI = confidence interval. AIC = Akaike Information Criterion.

**Table 2:** PGLS regression models on maximum absolute growth rate per day (AGR) against body mass at maximum growth (BMatMG, in gram) for different taxonomic groups assuming a fixed slope of 0.75.

group	N	intercept	95% CI	p-value	lambda	AIC	10^∧^intercept
altrical birds	343	−0.506	[−0.587, −0.425]	<2e-16	0.943	−524.679	0.312
precocial birds	164	−0.839	[−1.016, −0.663]	<2e-16	0.853	−185.665	0.145
eutherians	299	−1.162	[−1.306, −1.019]	<2e-16	0.826	−70.045	0.069
marsupials	21	−1.244	[−1.244, −1.244]	<2e-16	−0.124	−29.036	0.057
non-avian dinosaurs	19	−1.766	[−1.931, −1.602]	<2e-16	0.840	−16.541	0.017
reptiles	35	−2.160	[−2.461, −1.858]	<2e-16	0.222	51.923	0.007
fish	30	−2.512	[−3.048, −1.976]	<2e-16	0.839	13.890	0.003

AGR and BMatMG were log 10 transformed before regressions were conducted (log10 AGR = log10 intercept+log10 BMatMG * 0.75). Regression models are ordered by values of intercepts. N = sample size. CI = confidence interval. lambda = Pagel’s lambda. AIC = Akaike Information Criterion.

## Materials and Methods

### Data

We established phylogenetic generalized linear least square regressions (PGLS) on the logarithm (log 10) of maximum growth rate (AGR) and the logarithm of body mass at maximum growth (BMatMG) for altricial (N = 343) and precocial birds (N = 164, [11]). We only used bird species whose growth rates were derived from a Gompertz or logistic model. Development modes of birds were taken from Starck and Ricklefs [11] and were classified as follows: a and sa = altricial; p, sp, sp/p and su = precocial. For abbreviations see [11]. We analogously statistically analyzed eutherian mammals (N = 299, [12]), fishes (N = 30, [13]), reptiles (N = 35, [21]), and dinosaurs (N = 19, [21]). Since Zullinger [12] also provides growth rates for marsupials (N = 21) and his sample size is considerably larger than Case’s ([9], N = 4), we analysed this group, too. Phylogenetic trees, which were needed to control for shared evolutionary history of species, were adapted from literature and online databases ([22]–[27], for details see supporting information: Phylogenetic Information S1) to match the species of our datasets. To increase datasets, all names of species which did not match those in the respective phylogenetic tree were either corrected for typographical errors or the incorrect name(s) were updated when the taxonomy had changed using online databases ([28]–[31]).

For extant species, mean values for AGR and BMatMG were used when several values for the same species were available. For dinosaurs, we used all body masses and respective growth rates given in Griebeler [21]. With the exception of the maximum growth rates of the reptile species taken from Case’s study, all growth rates used were given as growth rate constants (k, [15]) of different growth models (Logistic, Gompertz, von Bertalanffy). If necessary, growth rate constants were converted to a daily basis. To make growth rates comparable between different growth models, we calculated maximum absolute growth rates (AGR, [15]) based on the fitted model: AGR = 1/2 * k_L_ * BMatMG, where *k_L_* is the growth rate constant from the logistic model, AGR = k_G_ * BMatMG, where *k_G_* is the growth rate constant from the Gompertz model and AGR = 3/2 * k_B_ * BMatMG, where *k_B_* is the growth rate constant from the von Bertalanffy model. To make the growth rates of reptiles taken from Case’s study comparable to the others, we assumed a von Bertalanffy model to estimate BMatMG. This model is often used for reptiles [2], [16], [32], [33] and is consistent with the determination of the growth rate in individuals done by Case. Case calculated growth rates in the interval of fastest absolute growth. In reptiles, he observed this period between week-old hatchlings until they reached 30% of total body mass. We used Case’s growth rates on reptiles, although they were not derived from fitted growth models, because fitted mass-specific growth models in reptiles are very rare in literature. All AGR and BMatMG values of the studied species are given in Table S1 in the supporting information.

### Statistical Analyses

#### Establishment of allometries for birds, mammals and reptiles

The first step taken was the separate analyses of the allometric relationships between AGR and body mass at maximum growth (BMatMG) of altricial birds, precocial birds, eutherian mammals, marsupials, reptiles and dinosaurs. For each of these taxa, we calculated regression slopes and normalization constants using PGLS in R (gls function, the ape package, and the Pagel correlation structure) on log-log-transformed data. Additionally, we calculated 95% confidence intervals (CI) for each regression coefficient. We also calculated ordinary least square regressions (OLS). This was done because the phylogenetic method reduced the sample sizes because no phylogenetic tree was available covering all species. Additionally, these results might also be useful for a comparison of the new regressions with other/older studies. For OLS results see Table S2, S3 and Figure S1.

In a second step, we tested whether or not the predicted value of 0.75 [5], [20] was in the 95% confidence interval of our calculated slopes. If the 0.75 was not statistically rejected, we calculated the normalization constants for each of the different taxonomic groups (Table 1 and 2) using a slope of 0.75 as a fixed value in our model. Therefore we used the gnls function [34] in R and the model log10 AGR ∼ intercept+log10 BMatMG * 0.75. Again we used the ape package and the Pagel correlation structure to control for phylogenetic effects. These regressions were then used to establish our allometries between AGR and body mass at maximum growth of the studied vertebrate groups (see Results). For our purpose, the fixed slope approach had an important advantage in comparison to “normal” PGLS. Deviation of a single sample because of a stochastic infelicitous sample composition did not have such a strong effect on the derived regression functions. For example, since regression coefficients (intercept and slope) are highly correlated with each other [35] (see also Table S4), regression models with different slopes and intercepts may describe data very well within a specific range. The exact coefficients could be strongly influenced by sample composition. Using a fixed slope to estimate normalization constants attenuates this effect. Altogether, this should yield more robust regressions. Furthermore, this approach conforms with the observation that, when relating body mass to other traits, normalization constants often differ between taxa, while equal or very similar slopes are observed between [36]–[39] or within taxa [40]–[42]. Finally, as a measure of variability in residuals and the deviation of single species from the expected average, we calculated for each regression line the residual variation as residuals+average (intercept) and compared these values between regression models (see also Results). The respective regression models were compared to dinosaur growth rates and to Case’s [10] regressions. All analyses and calculations were performed in R (Version 3.0.2, 64-bit, [43]).

## Results

### PGLS Regression Analyses

**Figure 1:**
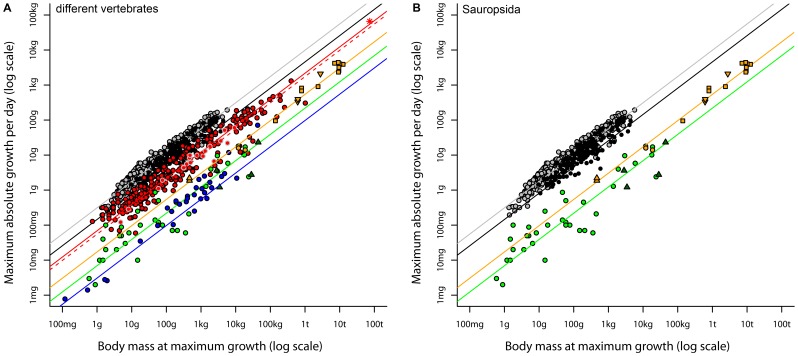
Regressions of maximum absolute growth rate (AGR) against body mass at maximum growth (BMatMG) for (A) different vertebrate groups and (B) for sauropsids only. Grey = altricial birds; black = precocial birds; red = mammals, red circles with black contour = eutherians, red circles with grey contour = marsupials, red star = blue whale; green = reptiles, dark green triangles = crocodiles; blue = fish; orange = dinosaurs, orange squares = sauropodomorphs, orange triangles upside down = theoropds, orange triangles = *Archaeopteryx*, orange circles = *Psittacosaurus*. Lines shown are regression lines with a slope forced to 0.75 (consistent with our empirical findings and with theory). For values of the intercepts see Table 2. Data on growth rate and body mass of the blue whale are from Case [9]. Please note that the blue whale was not used to establish our regression model on mammals. Also, crocodiles were not used to establish our regression model on reptiles. Crocodiles were not included in the phylogeny of reptiles, which was needed for PGLS.

There was a highly significant relationship between body mass at maximum growth (BMatMG) and maximum absolute growth rate (AGR) in all studied taxonomic groups (Table 1, see Table S2 for OLS analyses). On average the endothermic mammals and birds had higher growth rates than ectothermic reptiles and fishes (Table 1, see Table S2 for OLS analyses). The order of the intercepts of the extant taxa were equal to Case’s studies ([9], [10]) (altricial birds >precocial birds>eutherians >marsupials> reptiles>fishes, Figure 1A) and regression models of extant taxa differed statistically from each other (95% CI of intercept and/or slope does not include the intercept and/or slope of the other models). The intercept of the studied dinosaurs was intermediary to those of the marsupials and reptiles. However, dinosaurian intercept and slope were not statistically different from the reptile regression model (95% CI of the reptile intercept include the intercept of dinosaurs and 95% CI of the reptile slope include the slope of dinosaurs).

In general, PGLS slopes of all studied taxa coincide well with the value of 0.75 predicted by theory (also OLS slopes see Table S2). With the exception of altricial birds and fishes, the PGLS slopes of the studied groups did not differ statistically from 0.75 (Table 1). When using 0.75 as the fixed parameter (slope) in the PGLS regression model, however, all models including those of altricial birds and fishes were better than or equal to the regression models estimating both parameters (intercept and slope), as valued by the Akaike’s Information criterion (AIC, see [44]) (Table 1 and Table 2). The latter observation justifies the usage of a fixed slope for all vertebrate groups studied.

### Comparing Taxa using the Theoretically Predicted Value of 0.75 as the Fixed Parameter in the PGLS Regression Model

**Figure 2:**
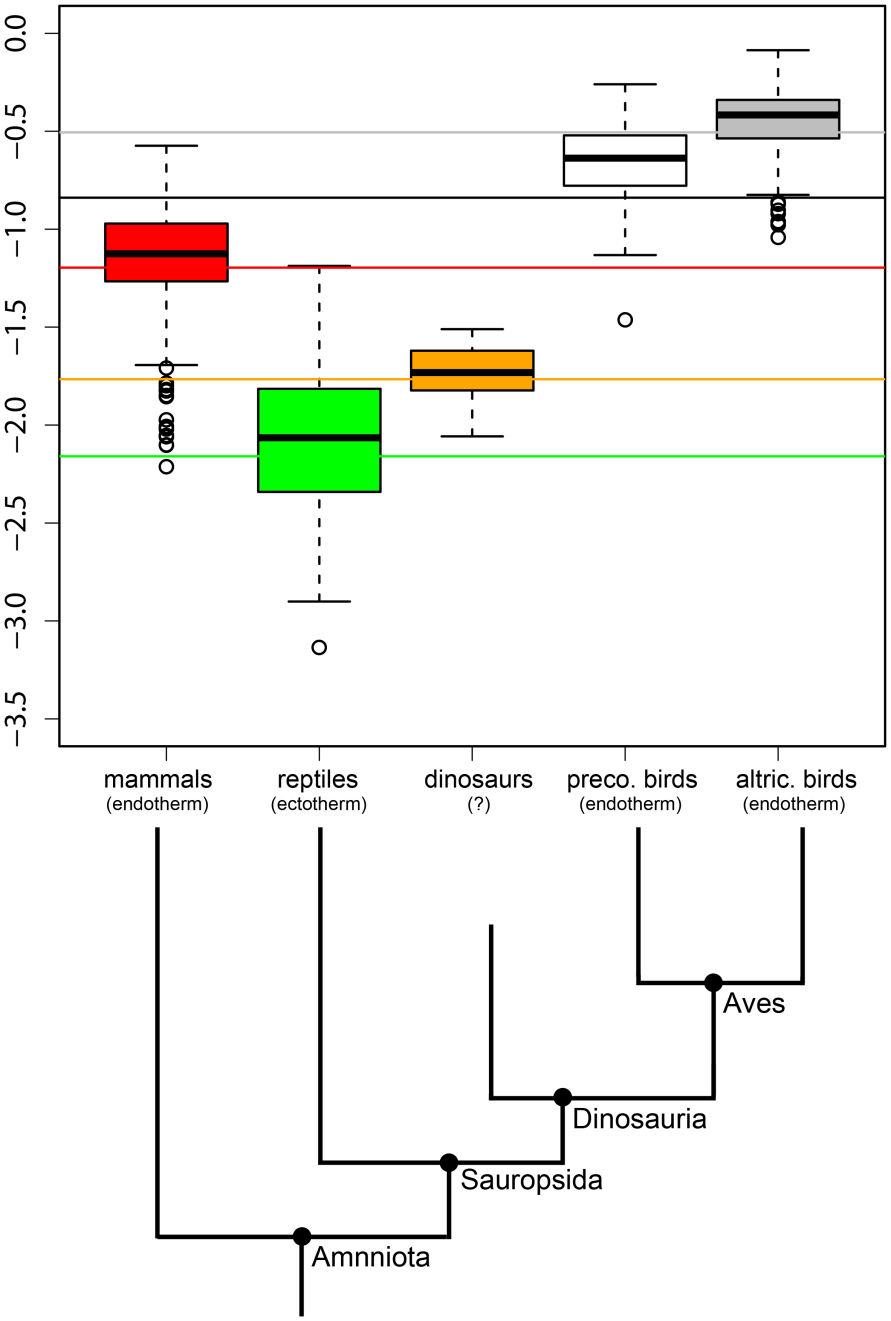
Comparison of the residual variation seen in maximum absolute growth rates of different vertebrate taxa. Taxa are ordered according to their phylogenetic relatedness. Presented are whisker-plots with means. The whiskers extend to the most extreme data point which is not more than 1.5 times (default value in R) the interquartile range given by the box. Open circles are outliers. Solid continuous lines represent the value of the intercept of the different amniotic groups (grey = altricial birds; black = precocial birds; red = mammals (eutherians+marsupials); green = reptiles; orange = dinosaurs). For values of the intercepts see Table 2.

When using 0.75 as the fixed parameter (slope) in the PGLS regression models, the same ranking of intercepts was observed as for the PGLS regression models estimating slopes and intercepts. The intercepts of most vertebrate groups differed significantly from each other (the intercept of most groups ranged outside the 95% CI of the intercept of any other group). Only the intercept of reptiles was in the 95% CI of fishes and the intercept of marsupials was in the 95% CI of eutherians (Table 2). While most intercepts differed between groups, the data points underlying the AGR to BMatMG relations overlapped between several groups (Figure 1A, Table 2). The individual AGR to BMatMG relations of ectotherms (reptiles and fishes) showed a stronger overlap (Figure 1 and 2) than with the endotherms (mammals and birds, Figure 1 and 2). Some individual ectothermic growth rates are similar to individual endothermic growth rates. Non-avian dinosaur growth rates overlapped with those of ectotherms (reptiles, fishes) and endotherms (mammals), but not with any endothermic recent dinosaurs (birds, Figure 1 and 2). Thus, within sauropsids the non-avian dinosaur growth rates fitted to growth rates of recent ectotherms, but not to those of recent endotherms (Figure 1B, Figure 2). Similar results were obtained for OLS regression analyses (see Figure S1).

### Comparing PGLS Regressions using 0.75 as the Fixed Parameter in the Model to Case Regressions [10]

**Figure 3:**
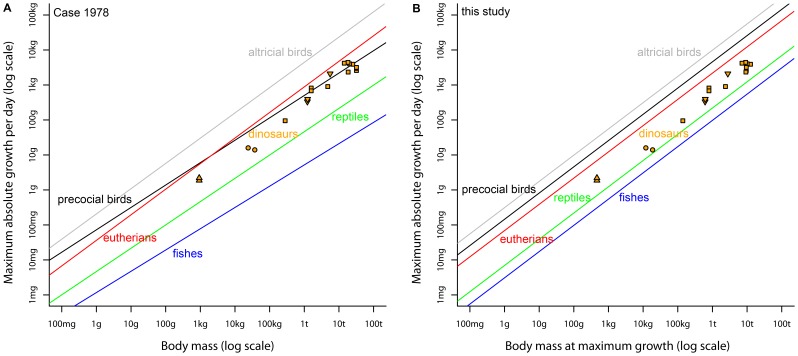
Comparison of (A) Case’s (1978) regression models with (B) our regression models with studied dinosaurian growth rates shown. Presented are the regression lines of altricial birds (black), precocial birds (grey), mammals (red), and reptiles (green). Orange points are non-avian dinosaurs: orange squares = sauropodomorphs, orange triangles upside down = theoropds, orange triangles = *Archaeopteryx*, orange circles = *Psittacosaurus*. For intercepts and slopes of regression models see Case (1978) and Table 2 (our models).

The theoretical slope of 0.75 was always higher than the slopes calculated by Case ([10]), (Figure 3). The greatest difference between Case’s slopes and the 0.75 was observed in precocial birds and fishes with a deviation of 15% or more, followed by reptiles with 11%. In contrast, Case’s slopes for altricial birds and eutherians differed by less than 4% from 0.75. Intercepts of reptiles and the regressions of fishes presented in Case did not statistically differ from our PGLS regressions with a fixed slope, whereas the intercept of the regressions for altricial birds, precocial birds and eutherians in Case’s study (1978) differed statistically from ours (Table 1 and 2). Nevertheless, the order of the magnitude of the intercepts for the different groups was identical (altricial birds >precocial birds>eutherians>reptiles>fishes, Figure 3). However, a plotting of dinosaurian growth rates into Case’s regressions and a comparison to our regressions (Figure 3) showed two remarkable differences between Case’s and our results. First, while dinosaurian growth rates, especially those of the larger dinosaurs, were well described by the precocial bird regression model of Case, our results clearly demonstrated that dinosaurian growth rates do not fit those of precocial birds (Figure 3). Second, dinosaurian growth rates are more different from ectothermic reptiles compared to Case’s reptile regression than compared to our reptile regression (Figure 3).

## Discussion

### PGLS Regression Analyses

With the exception of altricial birds and fishes, none of the slopes of the studied vertebrate groups statistically differed from 0.75, but they did statistically differ from 0.67 (except for reptiles and eutherians, Table 1). When using 0.75 as the fixed parameter (slope) in the PGLS regression models, all models including those of altricial birds and fishes were better than or equal to the regression models estimating both parameters (intercept and slope), as valued by AIC’s. Thus, our results corroborate the Metabolic Theory of Ecology (MTE), which predicts a slope of 0.75 for the allometry on maximum growth rate and body mass [5], [20]. Our results clearly refute a slope of 0.67, which is explained by the area to volume ratio of an animal [40]. However, it is beyond the scope of this paper to test the assumptions and predictions of the MTE or any other theoretical model on scaling of life history traits with body mass. Whatever the underlying mechanisms are, the accordance of our empirical findings with theoretical predictions and other empirical findings (e.g. [5], [20], [45]–[49]) strengthens our approach: using a slope of 0.75 as the fixed parameter in our regression models.

### Comparing Different Taxonomic Groups using the Theoretically Predicted Value of 0.75 as the Fixed Parameter in the Regression Model

Similar to Case’s study, our results revealed that growth rates differed between taxa. On average, ectothermic fishes and reptiles have lower growth rates than similar sized endothermic mammals or birds (our results, [10]). Maximum absolute growth rates of ectotherms are about 10 (reptiles) to 20 (fishes) times (in comparison to mammals) or even about 45 (reptiles) to 100 (fishes) times (in comparison to birds) lower than that of endotherms (Table 2). Thus, Case’s statement (i) that “the evolution of endothermy was a key factor in lifting physiological constrains upon growth rate” and Case’s statement (ii) that “the maximum observed growth rates of endotherms (except for some marsupials and anthropoid primates) are at least an order of magnitude greater than the maximum growth rate of any ectotherm” seem to be valid. However, there were also considerable differences in the average growth rates within ectotherms and in particular within endotherms. Reptiles have, on average, a two-fold higher growth rate than similar sized fishes. Birds have growth rates 2 to 4.5 times higher than mammals (Table 2). Thus, different thermoregulation strategies (ectothermy or endothermy) alone cannot explain differences in growth rates between taxa.

A highly controversial issue discussed is if and how metabolic rate and growth rate are linked to each other [5], [9], [10], [36], [47], [50]–[53]. Paleontologists are very interested in this issue. If the two rates are indeed related, bone histological studies of fossilized bones, which provide growth rate estimates [54]–[58] of taxa, may also give information on metabolic rate and thermoregulation in taxa. Thus, in the following discussion we will focus mainly on this aspect. Nevertheless, we are aware that parental care, food supply and the allocation of available energy to growth and various other demands of an organism could strongly influence its growth rate. When applicable, we will briefly discuss these aspects. Furthermore, we will not compare non-avian dinosaurs to fishes or marsupials due to the high agreement of the eutherian model with the marsupial data and the high agreement of the reptile model with the fish data (Figure 1).

The same order of intercepts of the studied taxonomic groups is observed for metabolic rates [37], [49], [59], [60] and growth rates. Additionally, similar slopes are observed in regression models of growth rate against body mass or metabolic rate against body mass [5], [9], [10], [12], [14], [20], [37], [49], [59]–[61]. Thus a link between growth rate and metabolic rate seems very likely. Consequently, the differences observed in growth rates might be caused by the different metabolic rates of the taxa. Metabolic rates of marsupials are 70–90% lower than in eutherians (McNab 1988) and this correlates well with our observed differences in AGRs between marsupials and eutherians (Table 2). However, comparing bird and mammal basal metabolic rates showed that they do not differ more than 1.5 times [35], [62], [63] on average, whereas AGRs in birds are 2 to 4.5 times higher than in mammals. Brown et al. [5] suggested that, excluding body mass, (internal) temperature is the main factor causing the differences in (basal) metabolic rates between similar-sized organisms of different taxa. Also, their model predicts only a 1.5 times higher metabolic rate (e^−E/(k*Tb_birds)^/e^−E/(k*Tb_mammals)^, E = 0.63, k = 8.6173324*10^−5^ (Boltzmann constant), Tb_birds = 314° Kelvin (41°C), Tb_mammals = 309° Kelvin (36°C)) for birds in comparison to mammals. Field metabolic rates vary only 1.2 (100 kg species) to two-fold (1 g species) between birds and mammals [60]. This suggests that metabolic rate alone cannot account for the differences seen between avian and mammalian growth rates. Physiological differences (e.g. different thermoregulation strategies [64], [65]) combined with differences in parental care (e.g. biparental care (feeding) in many birds [66], maternal care (lactation) in mammals [67]) during growth also contribute to the observed differences between birds and mammals (for detailed discussions on these aspects see [9], [21], [65]. The differences between fish and reptile growth rates might be mainly caused by different environmental temperatures alone; however, this could not be tested with our data on reptiles because temperatures were not measured in the studies on growth rates we selected (but see [21] for crocodiles).

Due to the strong temperature dependency of resting metabolic rates of ectotherms [5], [59], [68], a comparison of the resting metabolic rate of ectotherms with the basal metabolic rate of endotherms is not trivial. When comparing only the intercepts of regression models to determine differences in traits of different taxonomic groups, if the slopes differ considerably from each other further potential biases are present (see also [63]). To avoid the latter, we compared metabolic rates of reptiles, mammals and birds of different body masses (1 g and 100 kg species). Using Andrews’ [68] multiple regression model of reptile resting metabolic rates, which accounts for (ambient) temperature, revealed that a reptile’s metabolic rate (at 36°C) differs by more than 3 (100 kg species) to 9 (1 g species) times from that of a mammal (equation for mammals is from [35], Table 1, OLS regression, full tree). Comparing the resting metabolic rate of a reptile (at 40°C) with that of a bird showed that it is 3 to 7.5 (100 kg e.g. 1 g species, non-passerines) or 4 to 11 (100 kg e.g. 1 g species, passerines) times lower in reptiles (the equations used for birds are from [63], equations 3 and 4). Thus, even after adjusting for temperature, ectothermic resting metabolic rates are much lower than endothermic basal rates. Field metabolic rates of mammals are between 4 (100 kg species) to 25-fold (1 g species) higher than in reptiles, whereas field metabolic rates of birds are between 5 (100 kg species) and 54-fold (1 g species) higher than in reptiles. Growth rates of reptiles are about 10 times (mammals) or even 45 times (birds) lower than that of endotherms (Table 2). Therefore, it seems likely that metabolic rates (of adults) and growth rates are at least partly linked to each other.

However, metabolic rates and thermoregulation alone cannot explain all the differences identified between growth rates of ectotherms and endotherms, or in particular the full variability seen in growth rates (Figure 1 and 2). While the averages (regression lines) indicate a clear separation, individual growth rates overlapped between several taxa, even between endotherms and ectotherms (Figure 1 and 2). This indicates that an assignment of a species to ectotherms or endotherms solely based on its growth rate is not possible and that it is inappropriate to apply Case’s or our allometries at the single species level. Furthermore, intraspecific studies (see [50] and references therein) question the link between metabolic rates and growth. They demonstrate that the relationship between metabolic rates and growth could be positive, non-existing or even negative. This shows that other factors besides (basal/resting) metabolic rates or temperatures have a high influence on growth rates in organisms.

Another factor with a high influence on growth rate is food supply [14], [69], [70]. Burton et al. [50] proposed that when resources are abundant or predictable in availability, individuals with relatively high resting metabolic rates (RMR) can exhibit faster growth rates than low-RMR individuals. If these conditions are not met, individuals with high RMRs do not benefit from high growth rates or they experience lower rates of growth [50]. Furthermore, differences in the amount of parental care exist between extant ectotherms and endotherms, which probably contributes to some differences seen in growth of their offspring. However, it seems that parental care alone does not to have such a high impact on growth rates. Precocial birds showing, in general, a lower amount of parental care than mammals (because the latter wean their offspring after birth), have on average higher growth rates than mammals. Additionally, an identical partitioning of energy allocation to growth, maintenance and reproduction by an individual must not be warranted in different taxa and especially in ectotherms and endotherms.

To conclude, it is not possible to safely predict metabolic rates by comparing the growth rates of single organisms. Irrespective of which factors cause the differences in growth rates between extant birds and mammals or endotherms and ectotherms, our results and other studies show that endothermy or different metabolic rates could not per se explain the full variability seen in growth rates of vertebrates. Nevertheless, very high growth rates seem to be linked to a high (basal/resting) metabolic rate and a considerable level of parental care in extant organisms.

### Comparing Dinosaurian Growth Rates to Recent Vertebrate Growth Rates: were some Dinosaurs Endotherms?

What can we infer from growth rates of dinosaurs about their metabolism or thermoregulation strategy? Our results suggest that dinosaurian growth rates were between those predicted by the reptile and mammal regression model and that some dinosaurian growth rates were within the range of mammalian growth rates. Thus, one may argue that at least some dinosaurs were endotherms and/or had metabolic rates similar to those of mammals. However, some ectothermic growth rates are also consistent with the variability of growth rates of mammals (Figure 1 and 2). Growth rate to body mass relations of dinosaurs are located within the intersection of the ectothermic and endothermic growth rates (Figure 1 and 2). Given this, the interpretation of endothermy or ectothermy in dinosaurs would be ambiguous. However, restricted to sauropsids, all studied non-avian dinosaurian growth rate to body mass relations were in the range of reptile growth rates and did not fit within those of birds. Hence, they conform better to an ectothermic sauropsid model than to the recent endothermic dinosaurian model, the birds. Additionally, non-avian dinosaurian PGLS regression coefficients (intercept and slope) did not differ statistically from coefficients of the reptile regression model. All these arguments provide evidence that the studied dinosaurs had a lower metabolic rate than recent endotherms. Our findings are in agreement with McNab (2009), who inferred from field energetic expenditure arguments that dinosaurs had a metabolism similar to varanid lizards. Thus a careful interpretation of all these results suggests that dinosaurs probably had a higher average (basal/resting) metabolic rate than an average recent reptile. This is probably consistent with differences in anatomical features of dinosaurs and reptiles, like a straight gait and a complex lung structure, feathers or behavioral features found in extinct dinosaurs, but not in recent reptiles [71]–[75]. Furthermore, endothermy emerged in the dinosaurs [76], [77]. Thus dinosaurian species, which had not only intermediary morphological characters of ectotherms and endotherms but also physiological ones, should have lived. Seebacher [77] suggested that within the Dinosauria, the most likely candidate to have developed endothermy would be a small (feathered) dinosaur. The only small partly feathered dinosaur in our study, the *Archaeopteryx*, had growth rates similar to fast growing extant ectothermic reptiles and slow growing endothermic mammals (Figure 1, smallest dinosaur in the study). Based on Seebacher (2003), we suggest that the most likely candidate showing (probably) endothermy and a growth rate similar to an average recent mammal or recent bird would be a small (and feathered) dinosaur, which additionally provided considerable parental care to offspring (breeding and feeding their offspring). Large dinosaurs, like the sauropods, probably had limited (if at all) parental care during breeding and probably none after hatching, e.g. by feeding the young ([78], [79]). Therefore, high metabolic rates or endothermy as observed in recent mammals and birds seem to be very unlikely in sauropods (see also [80], [81]). Furthermore, the production of additional heat by a metabolic rate much higher than that of an ectotherm of similar size would have provided only small thermo-regulative advantages, if any at all, due to the gigantothermic effect in these large-bodied animals. In addition, large dinosaurs with a frugal metabolism because of a low mass-specific metabolic rate would not need so much food compared to a similar-sized endothermic species (see also McNab 2009). Thus, most large dinosaurs like the sauropods would probably not have actively regulated their body temperature by additional metabolic heat production at the same level as extant endotherms do.

### Comparison of Our Regressions to that of Case (1978)

Our results differ from those of other studies that compared dinosaurian growth rates to recent taxa using Case’s regressions. Why are there such strong differences between Case’s and our regressions when plotting dinosaurian growth rates? The most striking difference between Case’s and our regressions was observed for precocial birds (see Results, Figure 3). This has far reaching consequences when interpreting the level of growth rates of dinosaurs and their metabolism. Whereas under Case’s regression models dinosaurian growth rates, especially those of the larger dinosaurs, were well described by an endothermic dinosaurian (precocial bird) model, our results clearly demonstrated that none of the studied dinosaurian growth rates fit to those of endothermic dinosaurs (birds), but were in-between those of ectotherms and endotherms.

## Conclusion

The regression models introduced by Case [9], [10] for precocial birds and fishes should not be used anymore to compare growth rates of similar sized species, especially if these models are extrapolated in body mass. Due to the low sample sizes underlying these two models, they are biased. All models presented in this study are based on much larger sample sizes than the models given in Case [9], [10]. They are consistent with theoretical predictions and other empirical observations on scaling of maximum growth rate and metabolism in extant vertebrates. Additionally, some models on recent taxa (mammals, fishes) were established in a body mass range that is observed in some dinosaurian taxa. The blue whale, which was not used to establish our regression model for mammals, fitted well to the mammalian model. This aquatic mammal has a considerably larger body mass than the largest dinosaurs in our study, the sauropods, (Figure 1, data for the blue whale is from Case 1978). Our regression model on dinosaurian growth rates is consistent with the recent vertebrate models (Table 1). Thus, the regression models presented in this study are reliable and may provide the best models on growth rate and body mass at present, especially if growth rates of vertebrates are compared at a broad taxonomic or functional (ectothermy, endothermy) level.

Our regression models revealed that dinosaurs had growth rates intermediate to similar sized/scaled-up reptiles and mammals, but had much lower rates than those observed in scaled-up birds. Furthermore, our results suggest that – irrespective of whether all dinosaurian growth rates were higher than that of an average reptile - they were still in the range of rates seen in fast growing reptiles. Dinosaurian rates are also consistent with those of slow growing mammals, but no dinosaur had a growth rate consistent with any precocial or altricial bird. Thus, under the assumption that growth rate, metabolic rate and thermoregulation are directly linked, it is not possible to infer whether the studied dinosaurs had an ectothermic or endothermic metabolic rate because of the large variability seen in ectothermic and endothermic growth rates. However, compared to growth rates seen in other sauropsids, all studied dinosaurs had rather an ectothermic metabolic rate than an endothermic rate.

## Supporting Information

Figure S1 **Comparison of the residual variation seen in maximum absolute growth rates of different vertebrate taxa (OLS regression analyses).** Taxa are ordered according to their phylogenetic relatedness. Presented are whisker-plots with means. The whiskers extend to the most extreme data point which is not more than 1.5 times (default value in R) the interquartile range given by the box. Open circles are outliers. Solid continuous lines represent the value of the intercept of the different amniotic groups (grey = altricial birds; black = precocial birds; red = mammals (eutherians+marsupials); green = reptiles; orange = dinosaurs). For values of the intercepts see Table S3.
(TIF)

Table S1 **Data used in regression analyses of maximum absolute growth rate (AGR) vs body mass at maximum growth (BMatMG).** Species with asterix (*) were not within the phylogenies used to controll for phylogenetic effects and thus not used in the phylogenetic generelaized least square regression analyses (PGLS). BMatMG was calculated by multipling body mass with the percentage of growth at the point of inflection of the respective growth model (Gompertz = 1/*e*, logistic = 1/2, von Bertalanffy = 8/27). References of the data are given below the table. For further details see the Material and Method section in the manuscript. Relative growth rate (RGR) could calculated by dividing AGR through BMatMG.
(DOCX)

Table S2 **Ordinary linear least square (OLS) regression models of maximum absolute growth rate per day (AGR) on body mass at maximum growth (BMatMG, in gram) for different taxonomic groups.** AGR and BMatMG were log 10 transformed before OLS were performed, presented are the back transformed models (log10 AGR = log10 intercept+log10 BMatMG * slope). Models are ordered by values of intercepts. N = sample size. CI = confidence interval. AIC = Akaike Information Criterion.
(DOCX)

Table S3 **Ordinary linear least square (OLS) regression models of maximum absolute growth rate per day (AGR) on body mass at maximum growth (BMatMG, in gram) for different taxonomic groups but with a fixed slope of 0.75.** AGR and BMatMG were log 10 transformed before regressions were performed (log10 AGR = log10 intercept+log10 BMatMG * 0.75). Regression models are ordered by values of intercepts. N = sample size. CI = confidence interval. AIC = Akaike Information Criterion.
(DOCX)

Table S4 **Correlation of regression coefficients (intercept and slope) of the PGLS regression models.** For more information on the PGLS regression models see Table 2 in the manuscript.
(DOCX)

Phylogenetic Information S1 **Phylogenetic information used in PGLS regression analyses.** Newick format.
(DOCX)
