# Supplementary figures and images for: Allometries of Maximum Growth Rate versus Body Mass at Maximum Growth Indicate That Non-Avian Dinosaurs Had Growth Rates Typical of Fast Growing Ectothermic Sauropsids
Source: PLoS One. 2014 Feb 25;9(2):e88834. doi: 10.1371/journal.pone.0088834 (PMC3934860; doi:10.1371/journal.pone.0088834)

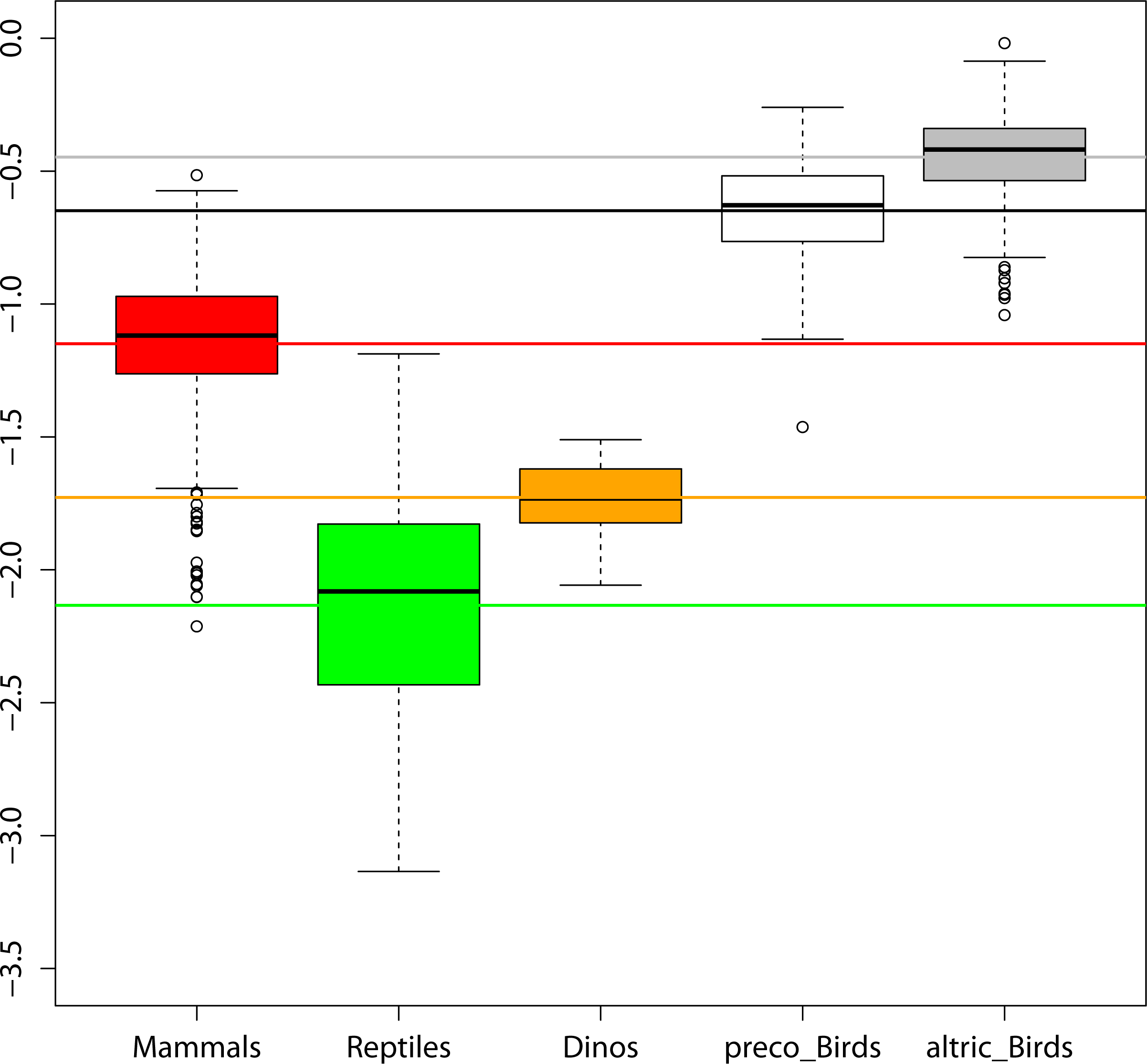

Supplement: Figure S1 — Comparison of the residual variation seen in maximum absolute growth rates of different vertebrate taxa (OLS regression analyses). Taxa are ordered according to their phylogenetic relatedness. Presented are whisker-plots with means. The whiskers extend to the most extreme data point which is not more than 1.5 times (default value in R) the interquartile range given by the box. Open circles are outliers. Solid continuous lines represent the value of the intercept of the different amniotic groups (grey = altricial birds; black = precocial birds; red = mammals (eutherians+marsupials); green = reptiles; orange = dinosaurs). For values of the intercepts see Table S3. (TIF) [file pone.0088834.s001.tif]
