# Supplementary material for: Allometries of Maximum Growth Rate versus Body Mass at Maximum Growth Indicate That Non-Avian Dinosaurs Had Growth Rates Typical of Fast Growing Ectothermic Sauropsids
Source: PLoS One. 2014 Feb 25;9(2):e88834. doi: 10.1371/journal.pone.0088834 (PMC3934860; doi:10.1371/journal.pone.0088834)
Supplement: Table S1 — Data used in regression analyses of maximum absolute growth rate (AGR) vs body mass at maximum growth (BMatMG). Species with asterix (*) were not within the phylogenies used to controll for phylogenetic effects and thus not used in the phylogenetic generelaized least square regression analyses (PGLS). BMatMG was calculated by multipling body mass with the percentage of growth at the point of inflection of the respective growth model (Gompertz = 1/e, logistic = 1/2, von Bertalanffy = 8/27). References of the data are given below the table. For further details see the Material and Method section in the manuscript. Relative growth rate (RGR) could calculated by dividing AGR through BMatMG. (DOCX) [file pone.0088834.s002.docx]

**Table S1**. Data used in regression analyses of maximum absolute growth rate (AGR) vs body mass at maximum growth (BMatMG). Species with asterix (*) were not within the phylogenies used to controll for phylogenetic effects and thus not used in the phylogenetic generelaized least square regression analyses (PGLS). BMatMG was calculated by multipling body mass with the percentage of growth at the point of inflection of the respective growth model (Gompertz = 1/*e*, logistic = 1/2, von Bertalanffy = 8/27). References of the data are given below the table. For further details see the Material and Method section in the manuscript. Relative growth rate (RGR) could calculated by dividing AGR through BMatMG.

| **Species** | | | | **AGR** | **BMatMG** |
| --- | --- | --- | --- | --- | --- |
| **Birds** | |  |  |  |  |
|  | **altricial birds** | | |  |  |
|  |  | **Apodiformes** | |  |  |
|  |  |  | *Amazilia fimbriata* | 0.256 | 2.000 |
|  |  |  | *Amazilia tobaci* | 0.349 | 2.100 |
|  |  |  | *Amazilia tzacatl* | 0.505 | 2.625 |
|  |  |  | *Apus apus* | 3.340 | 18.762 |
|  |  |  | *Apus pacificus* | 3.024 | 32.000 |
|  |  |  | *Calliphlox evelynae* | 0.337 | 1.650 |
|  |  |  | *Chaetura brachyura* | 1.671 | 10.250 |
|  |  |  | *Chaetura pelagica* | 2.070 | 12.000 |
|  |  |  | *Chaetura vauxi* | 2.100 | 10.500 |
|  |  |  | *Chlorostilbon mellisugus* | 0.195 | 1.400 |
|  |  |  | *Colibri coruscans* | 0.578 | 3.850 |
|  |  |  | *Collocalia esculenta* | 0.662 | 4.730 |
|  |  |  | *Collocalia fuciphaga* | 0.861 | 6.150 |
|  |  |  | *Collocalia leucophaea* | 0.346 | 4.650 |
|  |  |  | *Cypseloides cherriei* | 0.945 | 10.500 |
|  |  |  | *Cypseloides cryptus* | 1.698 | 17.500 |
|  |  |  | *Cypsiurus parvus* | 0.858 | 5.360 |
|  |  |  | *Lampornis clemenciae bessophilus** | 0.867 | 3.750 |
|  |  |  | *Oxypogon guerinii* | 0.317 | 2.725 |
|  |  |  | *Phaethornis ruber* | 0.317 | 1.350 |
|  |  |  | *Selasphorus rufus* | 0.336 | 1.768 |
|  |  |  | *Streptoprocne rutila* | 1.516 | 12.500 |
|  |  |  | *Tachymarptis melba* | 6.038 | 48.500 |
|  |  | **Caprimulgiformes** | |  |  |
|  |  |  | *Steatornis caripensis* | 14.072 | 290.367 |
|  |  | **Ciconiiformes** | |  |  |
|  |  |  | *Ardea cinerea* | 66.416 | 655.684 |
|  |  |  | *Ardea herodias* | 68.913 | 883.500 |
|  |  |  | *Ardea purpurea* | 42.085 | 356.650 |
|  |  |  | *Botaurus stellaris* | 46.800 | 399.834 |
|  |  |  | *Bubulcus ibis* | 16.582 | 151.250 |
|  |  |  | *Butorides virescens* | 10.448 | 73.576 |
|  |  |  | *Ciconia abdimii* | 45.900 | 600.000 |
|  |  |  | *Ciconia ciconia* | 125.375 | 1475.000 |
|  |  |  | *Ciconia maguari* | 120.299 | 1295.394 |
|  |  |  | *Egretta caerulea* | 19.350 | 150.000 |
|  |  |  | *Egretta garzetta* | 28.599 | 169.225 |
|  |  |  | *Eudocimus albus* | 24.355 | 383.453 |
|  |  |  | *Geronticus eremita* | 60.944 | 472.318 |
|  |  |  | *Leptoptilos crumeniferus* | 101.167 | 2023.337 |
|  |  |  | *Mycteria americana* | 78.174 | 919.699 |
|  |  |  | *Platalea leucorodia* | 71.775 | 825.000 |
|  |  |  | *Scopus umbretta* | 20.563 | 235.000 |
|  |  | **Coliiformes** | |  |  |
|  |  |  | *Urocolius macrourus* | 3.819 | 17.050 |
|  |  | **Columbiformes** | |  |  |
|  |  |  | *Columba guinea* | 17.259 | 135.900 |
|  |  |  | *Columba livia* | 22.278 | 137.681 |
|  |  |  | *Columba oenas* | 23.256 | 145.350 |
|  |  |  | *Columba palumbus* | 24.126 | 122.927 |
|  |  |  | *Columbina passerina* | 2.976 | 11.950 |
|  |  |  | *Columbina talpacoti* | 3.220 | 14.000 |
|  |  |  | *Geopelia cuneata* | 1.203 | 18.650 |
|  |  |  | *Oena capensis* | 1.175 | 17.400 |
|  |  |  | *Ptilinopus superbus* | 5.883 | 16.250 |
|  |  |  | *Streptopelia decaocto* | 11.173 | 49.222 |
|  |  | **Coraciiformes** | |  |  |
|  |  |  | *Coracias caudatus* | 4.669 | 37.500 |
|  |  |  | *Merops viridis* | 2.733 | 18.850 |
|  |  | **Cuculiformes** | |  |  |
|  |  |  | *Carpococcyx renauldi* | 12.000 | 200.000 |
|  |  |  | *Centropus senegalensis* | 12.488 | 67.500 |
|  |  |  | *Chrysococcyx basalis* | 2.240 | 10.925 |
|  |  |  | *Chrysococcyx caprius* | 3.394 | 18.100 |
|  |  |  | *Chrysococcyx lucidus* | 1.947 | 11.125 |
|  |  |  | *Clamator glandarius* | 11.261 | 70.125 |
|  |  |  | *Crotophaga ani* | 5.897 | 17.950 |
|  |  | **Falconiformes** | |  |  |
|  |  |  | *Accipiter cooperii* | 19.753 | 187.333 |
|  |  |  | *Accipiter fasciatus* | 23.074 | 221.516 |
|  |  |  | *Accipiter gentilis* | 57.122 | 508.000 |
|  |  |  | *Accipiter minullus* | 6.481 | 42.500 |
|  |  |  | *Accipiter nisus* | 13.596 | 106.625 |
|  |  |  | *Aquila chrysaetos* | 113.241 | 1670.942 |
|  |  |  | *Aquila rapax* | 60.450 | 1300.000 |
|  |  |  | *Aquila verreauxii* | 77.524 | 1649.440 |
|  |  |  | *Aquila wahlbergi* | 26.303 | 404.667 |
|  |  |  | *Buteo buteo* | 43.092 | 456.000 |
|  |  |  | *Buteo jamaicensis* | 48.595 | 523.750 |
|  |  |  | *Buteo oreophilus* | 26.495 | 298.775 |
|  |  |  | *Buteo platypterus* | 19.637 | 145.500 |
|  |  |  | *Buteo regalis* | 52.283 | 611.500 |
|  |  |  | *Buteo swainsoni* | 28.045 | 355.000 |
|  |  |  | *Cathartes aura* | 46.211 | 733.500 |
|  |  |  | *Circaetus cinereus* | 19.425 | 1050.000 |
|  |  |  | *Circus cyaneus* | 27.263 | 221.600 |
|  |  |  | *Circus ranivorus* | 31.343 | 220.728 |
|  |  |  | *Coragyps atratus* | 45.462 | 851.296 |
|  |  |  | *Falco cenchroides* | 13.500 | 100.000 |
|  |  |  | *Falco chicquera* | 12.738 | 112.750 |
|  |  |  | *Falco columbarius* | 13.919 | 105.075 |
|  |  |  | *Falco peregrinus* | 42.398 | 435.500 |
|  |  |  | *Falco sparverius* | 9.623 | 66.145 |
|  |  |  | *Falco subbuteo* | 15.334 | 120.167 |
|  |  |  | *Falco tinnunculus* | 18.486 | 125.250 |
|  |  |  | *Gyps africanus* | 69.100 | 2700.018 |
|  |  |  | *Gyps coprotheres* | 122.490 | 4149.667 |
|  |  |  | *Haliaeetus albicilla* | 155.562 | 1911.820 |
|  |  |  | *Haliaeetus leucocephalus* | 112.436 | 1904.323 |
|  |  |  | *Haliaeetus vocifer* | 89.580 | 1006.518 |
|  |  |  | *Hieraaetus fasciatus** | 46.200 | 600.000 |
|  |  |  | *Ictinia mississippiensis* | 11.830 | 140.000 |
|  |  |  | *Lophaetus occipitalis* | 44.213 | 675.000 |
|  |  |  | *Milvus migrans* | 40.938 | 410.653 |
|  |  |  | *Necrosyrtes monachus* | 35.510 | 1060.000 |
|  |  |  | *Neophron percnopterus* | 52.000 | 800.000 |
|  |  |  | *Pandion haliaetus* | 67.380 | 786.050 |
|  |  |  | *Sagittarius serpentarius* | 77.479 | 1597.500 |
|  |  |  | *Sarcoramphus papa* | 50.934 | 1306.000 |
|  |  |  | *Stephanoaetus coronatus* | 25.813 | 1475.000 |
|  |  |  | *Terathopius ecaudatus* | 34.125 | 1050.000 |
|  |  |  | *Torgos tracheliotus** | 114.075 | 3510.000 |
|  |  |  | *Trigonoceps occipitalis* | 75.568 | 2361.500 |
|  |  |  | *Vultur gryphus* | 120.393 | 3087.000 |
|  |  | **Opisthocomiformes** | |  |  |
|  |  |  | *Opisthocomus hoazin* | 8.213 | 174.743 |
|  |  | **Passeriformes** | |  |  |
|  |  |  | *Acrocephalus arundinaceus* | 3.412 | 12.900 |
|  |  |  | *Acrocephalus scirpaceus* | 1.553 | 5.825 |
|  |  |  | *Agelaius phoeniceus* | 4.967 | 17.875 |
|  |  |  | *Agelaius xanthomus* | 2.906 | 10.926 |
|  |  |  | *Aimophila carpalis* | 1.771 | 6.150 |
|  |  |  | *Alauda arvensis* | 3.630 | 12.000 |
|  |  |  | *Amandava amandava* | 0.688 | 3.600 |
|  |  |  | *Ammodramus aurifrons* | 1.650 | 6.483 |
|  |  |  | *Ammodramus caudacutus* | 2.100 | 7.500 |
|  |  |  | *Ammodramus maritimus* | 2.176 | 8.000 |
|  |  |  | *Amphispiza belli* | 2.021 | 8.250 |
|  |  |  | *Anthus spinoletta* | 2.486 | 9.600 |
|  |  |  | *Aphelocoma coerulescens* | 5.025 | 30.000 |
|  |  |  | *Arremonops conirostris* | 3.851 | 16.600 |
|  |  |  | *Bombycilla cedrorum* | 4.550 | 17.500 |
|  |  |  | *Buphagus erythrorhynchus* | 5.384 | 32.050 |
|  |  |  | *Calcarius lapponicus* | 3.462 | 11.400 |
|  |  |  | *Calcarius mccownii* | 2.356 | 9.500 |
|  |  |  | *Campylorhynchus brunneicapillus* | 3.093 | 15.700 |
|  |  |  | *Cardinalis cardinalis* | 4.128 | 16.000 |
|  |  |  | *Carduelis flammea* | 1.632 | 6.000 |
|  |  |  | *Carduelis spinus* | 1.287 | 6.500 |
|  |  |  | *Catharus fuscescens* | 3.600 | 15.000 |
|  |  |  | *Catharus guttatus* | 3.807 | 13.625 |
|  |  |  | *Catharus minimus* | 3.519 | 11.500 |
|  |  |  | *Catharus ustulatus* | 4.524 | 14.500 |
|  |  |  | *Cercomacra tyrannina* | 1.905 | 7.150 |
|  |  |  | *Certhilauda albescens* | 1.853 | 10.650 |
|  |  |  | *Chasiempis sandwichensis* | 1.616 | 7.750 |
|  |  |  | *Cistothorus palustris* | 1.218 | 6.000 |
|  |  |  | *Corvus albus* | 35.700 | 262.500 |
|  |  |  | *Corvus brachyrhynchos* | 19.350 | 225.000 |
|  |  |  | *Corvus capensis* | 37.295 | 265.975 |
|  |  |  | *Corvus caurinus* | 22.723 | 152.500 |
|  |  |  | *Corvus cornix** | 23.215 | 192.750 |
|  |  |  | *Corvus corone* | 31.537 | 193.500 |
|  |  |  | *Corvus corone corone** | 25.300 | 195.400 |
|  |  |  | *Corvus cryptoleucus* | 28.350 | 225.000 |
|  |  |  | *Corvus monedula* | 15.216 | 100.630 |
|  |  |  | *Cyanerpes cyaneus* | 0.840 | 6.000 |
|  |  |  | *Cyanocorax beecheii* | 11.691 | 64.950 |
|  |  |  | *Delichon urbicum* | 2.024 | 12.067 |
|  |  |  | *Dendroica kirtlandii* | 1.966 | 6.850 |
|  |  |  | *Dendroica petechia* | 2.540 | 12.450 |
|  |  |  | *Elaenia chiriquensis* | 1.357 | 5.850 |
|  |  |  | *Elaenia flavogaster* | 1.406 | 9.500 |
|  |  |  | *Empidonax difficilis* | 1.210 | 5.550 |
|  |  |  | *Empidonax traillii* | 1.770 | 7.500 |
|  |  |  | *Eremophila alpestris* | 3.381 | 13.125 |
|  |  |  | *Eremophila alpestris praticola** | 3.338 | 10.350 |
|  |  |  | *Erithacus rubecula* | 2.173 | 10.125 |
|  |  |  | *Euphagus cyanocephalus* | 5.405 | 23.050 |
|  |  |  | *Euphonia laniirostris* | 1.287 | 7.550 |
|  |  |  | *Euphonia minuta* | 0.710 | 5.000 |
|  |  |  | *Euphonia violacea* | 1.668 | 7.250 |
|  |  |  | *Ficedula hypoleuca* | 1.809 | 7.113 |
|  |  |  | *Foudia aldabrana** | 2.309 | 11.515 |
|  |  |  | *Geothlypis trichas* | 1.575 | 5.250 |
|  |  |  | *Gerygone igata* | 0.765 | 3.680 |
|  |  |  | *Gymnorhinus cyanocephalus* | 6.136 | 40.500 |
|  |  |  | *Hirundo rustica* | 2.652 | 10.924 |
|  |  |  | *Hirundo tahitica* | 1.722 | 8.200 |
|  |  |  | *Hylocichla mustelina* | 4.797 | 19.500 |
|  |  |  | *Lanius collurio* | 3.042 | 13.450 |
|  |  |  | *Lanius ludovicianus* | 5.400 | 24.000 |
|  |  |  | *Leucosticte tephrocotis* | 4.263 | 21.000 |
|  |  |  | *Lonchura oryzivora** | 2.053 | 11.500 |
|  |  |  | *Lonchura striata* | 0.509 | 4.650 |
|  |  |  | *Loxioides bailleui* | 2.769 | 17.250 |
|  |  |  | *Margarops fuscatus* | 9.133 | 44.247 |
|  |  |  | *Melampitta lugubris* | 1.881 | 19.000 |
|  |  |  | *Melospiza melodia* | 2.420 | 10.000 |
|  |  |  | *Microbates collaris* | 1.148 | 4.450 |
|  |  |  | *Mimus polyglottos* | 4.418 | 19.550 |
|  |  |  | *Mionectes macconnelli* | 1.044 | 7.250 |
|  |  |  | *Molothrus ater* | 4.320 | 15.000 |
|  |  |  | *Motacilla cinerea* | 2.763 | 9.150 |
|  |  |  | *Myadestes ralloides* | 3.083 | 15.300 |
|  |  |  | *Myiobius atricaudus* | 0.870 | 5.000 |
|  |  |  | *Myioborus miniatus* | 1.214 | 4.650 |
|  |  |  | *Myiodynastes maculatus* | 3.427 | 17.850 |
|  |  |  | *Myiozetetes cayanensis* | 2.266 | 11.500 |
|  |  |  | *Myiozetetes similis* | 1.951 | 12.750 |
|  |  |  | *Myrmeciza exsul* | 2.620 | 10.000 |
|  |  |  | *Myrmecocichla formicivora* | 4.883 | 21.850 |
|  |  |  | *Myrmotherula axillaris* | 0.861 | 4.200 |
|  |  |  | *Nectarinia jugularis* | 0.796 | 4.300 |
|  |  |  | *Nectarinia osea* | 0.738 | 3.100 |
|  |  |  | *Nectarinia talatala* | 0.863 | 4.250 |
|  |  |  | *Nucifraga caryocatactes* | 11.690 | 83.500 |
|  |  |  | *Oenanthe oenanthe* | 2.690 | 11.200 |
|  |  |  | *Parus ater* | 0.918 | 5.100 |
|  |  |  | *Parus atricapillus* | 1.380 | 5.750 |
|  |  |  | *Parus caeruleus* | 1.084 | 5.383 |
|  |  |  | *Parus major* | 1.764 | 9.072 |
|  |  |  | *Parus montanus* | 1.183 | 5.830 |
|  |  |  | *Parus palustris* | 0.986 | 5.600 |
|  |  |  | *Passer domesticus* | 2.577 | 12.267 |
|  |  |  | *Passerculus sandwichensis* | 2.272 | 7.700 |
|  |  |  | *Passerculus sandwichensis princeps** | 3.900 | 12.150 |
|  |  |  | *Petrochelidon ariel** | 1.391 | 4.635 |
|  |  |  | *Petrochelidon pyrrhonota* | 3.059 | 12.333 |
|  |  |  | *Petrochelidon spilodera** | 2.550 | 12.390 |
|  |  |  | *Phoenicurus phoenicurus* | 2.137 | 8.063 |
|  |  |  | *Phylloscopus collybita* | 1.236 | 4.963 |
|  |  |  | *Phylloscopus sibilatrix* | 1.500 | 5.640 |
|  |  |  | *Phylloscopus trochilus* | 1.373 | 4.785 |
|  |  |  | *Pica pica* | 12.919 | 90.750 |
|  |  |  | *Pipilo aberti* | 3.903 | 16.400 |
|  |  |  | *Pipilo erythrophthalmus* | 3.660 | 15.000 |
|  |  |  | *Pipra mentalis* | 0.914 | 5.050 |
|  |  |  | *Platycichla flavipes** | 5.113 | 25.000 |
|  |  |  | *Plectrophenax nivalis* | 4.464 | 15.500 |
|  |  |  | *Pogonocichla stellata* | 2.325 | 10.275 |
|  |  |  | *Pomatostomus temporalis* | 4.970 | 28.000 |
|  |  |  | *Pooecetes gramineus* | 2.978 | 9.550 |
|  |  |  | *Procnias averano* | 2.835 | 45.000 |
|  |  |  | *Progne chalybea* | 4.069 | 20.600 |
|  |  |  | *Progne subis* | 5.760 | 30.000 |
|  |  |  | *Psarocolius wagleri* | 6.747 | 51.503 |
|  |  |  | *Pygochelidon cyanoleuca* | 0.980 | 5.000 |
|  |  |  | *Pyrrhocorax graculus* | 14.857 | 106.500 |
|  |  |  | *Pyrrhocorax pyrrhocorax* | 23.513 | 171.000 |
|  |  |  | *Pyrrhomyias cinnamomeus* | 0.815 | 5.000 |
|  |  |  | *Quiscalus major* | 8.047 | 41.925 |
|  |  |  | *Quiscalus mexicanus* | 8.663 | 45.000 |
|  |  |  | *Quiscalus mexicanus prosopidicola** | 9.945 | 36.972 |
|  |  |  | *Quiscalus niger* | 3.589 | 17.050 |
|  |  |  | *Ramphocelus carbo* | 2.646 | 10.500 |
|  |  |  | *Ramsayornis modestus* | 1.311 | 5.800 |
|  |  |  | *Regulus ignicapilla* | 1.225 | 4.400 |
|  |  |  | *Regulus regulus* | 0.685 | 3.115 |
|  |  |  | *Rhynchocyclus olivaceus* | 1.689 | 9.650 |
|  |  |  | *Sakesphorus canadensis* | 1.570 | 10.000 |
|  |  |  | *Saltator albicollis* | 2.687 | 13.500 |
|  |  |  | *Saxicola rubetra* | 2.212 | 10.215 |
|  |  |  | *Saxicola rubicola** | 1.909 | 7.453 |
|  |  |  | *Saxicola torquatus* | 2.188 | 7.985 |
|  |  |  | *Saxicola torquatus axillaris** | 2.471 | 9.100 |
|  |  |  | *Seiurus aurocapilla* | 2.112 | 8.000 |
|  |  |  | *Seiurus motacilla* | 2.376 | 9.000 |
|  |  |  | *Sericulus chrysocephalus* | 2.852 | 46.000 |
|  |  |  | *Serinus serinus* | 0.788 | 5.000 |
|  |  |  | *Setophaga aestiva** | 2.905 | 9.950 |
|  |  |  | *Setophaga ruticilla* | 1.377 | 4.050 |
|  |  |  | *Sialia sialis* | 3.328 | 13.750 |
|  |  |  | *Spizella breweri* | 1.444 | 5.300 |
|  |  |  | *Spizella passerina* | 1.478 | 5.438 |
|  |  |  | *Spizella pusilla* | 1.709 | 5.500 |
|  |  |  | *Sporophila castaneiventris* | 0.834 | 3.700 |
|  |  |  | *Sporophila corvina* | 0.940 | 4.325 |
|  |  |  | *Sporophila nigricollis* | 0.926 | 3.875 |
|  |  |  | *Stelgidopteryx ruficollis* | 2.128 | 9.250 |
|  |  |  | *Sturnus vulgaris* | 7.745 | 37.886 |
|  |  |  | *Sylvia atricapilla* | 1.514 | 6.315 |
|  |  |  | *Sylvia borin* | 1.001 | 8.947 |
|  |  |  | *Tachycineta albilinea* | 1.573 | 7.750 |
|  |  |  | *Tachycineta bicolor* | 2.503 | 10.875 |
|  |  |  | *Tachycineta thalassina* | 2.215 | 10.750 |
|  |  |  | *Taeniopygia guttata* | 0.791 | 6.200 |
|  |  |  | *Tangara inornata* | 2.016 | 9.000 |
|  |  |  | *Thamnophilus doliatus* | 1.967 | 11.850 |
|  |  |  | *Thraupis episcopus* | 2.316 | 12.775 |
|  |  |  | *Thraupis palmarum* | 2.749 | 14.375 |
|  |  |  | *Todirostrum cinereum* | 0.707 | 3.600 |
|  |  |  | *Tolmomyias sulphurescens* | 1.269 | 8.750 |
|  |  |  | *Toxostoma curvirostre* | 5.726 | 27.650 |
|  |  |  | *Toxostoma longirostre* | 5.526 | 24.950 |
|  |  |  | *Troglodytes aedon* | 1.299 | 5.600 |
|  |  |  | *Troglodytes musculus** | 1.638 | 7.000 |
|  |  |  | *Troglodytes troglodytes* | 1.086 | 4.600 |
|  |  |  | *Turdus grayi* | 6.158 | 28.200 |
|  |  |  | *Turdus leucomelas* | 7.357 | 34.950 |
|  |  |  | *Turdus merula* | 7.735 | 32.500 |
|  |  |  | *Turdus migratorius* | 7.470 | 30.000 |
|  |  |  | *Turdus pilaris* | 12.226 | 36.700 |
|  |  |  | *Turdus torquatus alpestris** | 11.075 | 26.119 |
|  |  |  | *Tyrannus forficatus* | 3.014 | 15.300 |
|  |  |  | *Tyrannus melancholicus* | 2.662 | 19.150 |
|  |  |  | *Tyrannus tyrannus* | 3.624 | 16.106 |
|  |  |  | *Tyrannus verticalis* | 3.744 | 18.000 |
|  |  |  | *Vireo flavoviridis* | 2.010 | 7.500 |
|  |  |  | *Volatinia jacarina* | 0.992 | 4.700 |
|  |  |  | *Xanthocephalus xanthocephalus* | 6.240 | 24.370 |
|  |  |  | *Zonotrichia albicollis* | 2.968 | 9.488 |
|  |  |  | *Zonotrichia atricapilla* | 4.108 | 13.000 |
|  |  |  | *Zonotrichia capensis* | 2.023 | 9.114 |
|  |  |  | *Zonotrichia leucophrys* | 2.624 | 10.250 |
|  |  |  | *Zonotrichia leucophrys oriantha** | 3.323 | 10.400 |
|  |  | **Pelecaniformes** | |  |  |
|  |  |  | *Fregata aquila* | 12.416 | 459.849 |
|  |  |  | *Fregata ariel* | 8.540 | 427.000 |
|  |  |  | *Fregata magnificens* | 14.255 | 584.382 |
|  |  |  | *Fregata minor* | 17.472 | 505.282 |
|  |  |  | *Morus bassanus* | 94.700 | 1484.874 |
|  |  |  | *Morus serrator* | 62.744 | 1140.794 |
|  |  |  | *Phalacrocorax aristotelis* | 70.713 | 829.033 |
|  |  |  | *Phalacrocorax auritus* | 70.582 | 838.584 |
|  |  |  | *Phalacrocorax carbo* | 71.736 | 919.699 |
|  |  |  | *Sula dactylatra* | 39.394 | 842.543 |
|  |  |  | *Sula leucogaster* | 41.386 | 533.425 |
|  |  |  | *Sula sula* | 13.915 | 366.798 |
|  |  | **Phaethontiformes** | |  |  |
|  |  |  | *Phaethon aethereus* | 17.658 | 294.304 |
|  |  |  | *Phaethon lepturus* | 10.121 | 145.717 |
|  |  |  | *Phaethon rubricauda* | 19.884 | 312.698 |
|  |  | **Piciformes** | |  |  |
|  |  |  | *Dendrocopos leucotos* | 6.622 | 41.000 |
|  |  |  | *Dendrocopos major* | 7.538 | 37.500 |
|  |  |  | *Dryocopus martius martius** | 19.499 | 126.667 |
|  |  |  | *Dryocopus pileatus* | 16.599 | 88.291 |
|  |  |  | *Jynx torquilla torquilla** | 2.611 | 14.000 |
|  |  |  | *Picoides borealis* | 3.521 | 20.113 |
|  |  |  | *Picus canus canus** | 9.928 | 54.550 |
|  |  |  | *Ramphastos dicolorus* | 14.925 | 150.000 |
|  |  | **Procellariiformes** | |  |  |
|  |  |  | *Pelecanus occidentalis* | 104.301 | 1610.759 |
|  |  |  | *Pelecanus rufescens* | 165.969 | 2937.500 |
|  |  | **Psittaciformes** | |  |  |
|  |  |  | *Agapornis personatus* | 2.820 | 24.000 |
|  |  |  | *Agapornis roseicollis* | 3.003 | 26.000 |
|  |  |  | *Amazona aestiva* | 18.272 | 201.900 |
|  |  |  | *Amazona dufresniana* | 30.958 | 305.000 |
|  |  |  | *Amazona finschi* | 16.224 | 174.450 |
|  |  |  | *Amazona ochrocephala* | 23.570 | 298.350 |
|  |  |  | *Amazona ochrocephala ochrocephala** | 18.443 | 223.550 |
|  |  |  | *Amazona oratrix* | 18.561 | 236.450 |
|  |  |  | *Amazona viridigenalis* | 16.395 | 167.300 |
|  |  |  | *Ara ambiguus ambiguus** | 40.882 | 687.100 |
|  |  |  | *Ara ararauna* | 35.163 | 558.150 |
|  |  |  | *Ara chloropterus* | 35.781 | 638.950 |
|  |  |  | *Ara macao* | 34.065 | 567.750 |
|  |  |  | *Ara militaris* | 28.428 | 473.800 |
|  |  |  | *Ara rubrogenys* | 17.160 | 264.000 |
|  |  |  | *Bolborhynchus lineola* | 3.413 | 31.600 |
|  |  |  | *Cacatua alba* | 20.355 | 313.150 |
|  |  |  | *Cacatua galerita triton** | 24.305 | 385.800 |
|  |  |  | *Cacatua haematuropygia* | 11.772 | 181.100 |
|  |  |  | *Cacatua sanguinea* | 16.096 | 195.100 |
|  |  |  | *Cacatua sulphurea* | 14.445 | 209.350 |
|  |  |  | *Calyptorhynchus funereus* | 17.930 | 212.211 |
|  |  |  | *Eclectus roratus vosmaeri** | 13.869 | 216.700 |
|  |  |  | *Enicognathus ferrugineus* | 8.368 | 93.500 |
|  |  |  | *Eolophus roseicapilla** | 14.086 | 138.687 |
|  |  |  | *Forpus passerinus* | 1.392 | 11.850 |
|  |  |  | *Melopsittacus undulatus* | 2.426 | 21.000 |
|  |  |  | *Myiopsitta monachus* | 6.338 | 51.950 |
|  |  |  | *Primolius auricollis* | 11.946 | 148.400 |
|  |  |  | *Prosopeia tabuensis* | 11.203 | 138.500 |
|  |  |  | *Psittacus erithacus erithacus** | 15.142 | 236.600 |
|  |  |  | *Psittinus cyanurus cyanurus** | 2.325 | 37.500 |
|  |  | **Sphenisciformes** | |  |  |
|  |  |  | *Aptenodytes forsteri* | 193.872 | 5702.131 |
|  |  |  | *Aptenodytes patagonicus* | 155.000 | 2500.000 |
|  |  |  | *Eudyptes chrysocome* | 61.804 | 1030.062 |
|  |  |  | *Eudyptes pachyrhynchus* | 56.145 | 1477.500 |
|  |  |  | *Eudyptula minor iredalei** | 25.359 | 321.000 |
|  |  |  | *Eudyptula minor minor** | 35.538 | 575.500 |
|  |  |  | *Eudyptula minor novaehollandiae** | 31.530 | 454.088 |
|  |  |  | *Eudyptula minor variabilis** | 34.133 | 555.000 |
|  |  |  | *Megadyptes antipodes* | 100.194 | 2133.258 |
|  |  |  | *Pygoscelis adeliae* | 127.042 | 1644.055 |
|  |  |  | *Pygoscelis antarcticus* | 131.753 | 2001.431 |
|  |  |  | *Pygoscelis papua* | 134.930 | 2722.737 |
|  |  |  | *Spheniscus demersus* | 62.574 | 1687.482 |
|  |  |  | *Spheniscus magellanicus* | 43.551 | 982.022 |
|  |  | **Strigiformes** | |  |  |
|  |  |  | *Asio flammeus galapagoensis** | 8.306 | 123.975 |
|  |  |  | *Asio otus* | 13.950 | 124.000 |
|  |  |  | *Bubo bubo* | 70.730 | 940.334 |
|  |  |  | *Bubo lacteus* | 37.140 | 571.390 |
|  |  |  | *Bubo scandiacus** | 63.165 | 625.395 |
|  |  |  | *Bubo virginianus* | 41.497 | 441.455 |
|  |  |  | *Megascops asio* | 7.920 | 60.000 |
|  |  |  | *Otus senegalensis* | 3.630 | 31.700 |
|  |  |  | *Strix uralensis* | 18.999 | 263.034 |
|  |  |  | *Tyto alba* | 21.024 | 269.015 |
|  |  |  | *Tyto alba pratincola** | 20.864 | 256.000 |
|  |  |  | *Tyto javanica** | 19.495 | 307.000 |
|  |  |  |  |  |  |
|  | **precocial birds** | | |  |  |
|  |  | **Anseriformes** | |  |  |
|  |  |  | *Aix galericulata* | 9.867 | 210.611 |
|  |  |  | *Anas platyrhynchos* | 25.170 | 369.743 |
|  |  |  | *Anas platyrhynchos conboschas** | 40.685 | 568.000 |
|  |  |  | *Anas rubripes* | 23.590 | 413.864 |
|  |  |  | *Anas strepera* | 18.651 | 239.122 |
|  |  |  | *Anser anser* | 81.783 | 1164.338 |
|  |  |  | *Anser caerulescens** | 53.508 | 1372.000 |
|  |  |  | *Anser indicus* | 35.100 | 1300.000 |
|  |  |  | *Aythya affinis* | 12.385 | 199.759 |
|  |  |  | *Aythya americana* | 18.600 | 353.634 |
|  |  |  | *Aythya fuligula* | 20.400 | 283.125 |
|  |  |  | *Aythya valisineria* | 19.795 | 335.506 |
|  |  |  | *Branta hutchinsii minima** | 36.423 | 471.621 |
|  |  |  | *Branta nigricans** | 25.029 | 455.067 |
|  |  |  | *Branta nigricans orientalis** | 39.781 | 636.500 |
|  |  |  | *Chloephaga picta* | 49.407 | 574.500 |
|  |  |  | *Cygnus olor* | 116.297 | 2888.750 |
|  |  |  | *Dendrocygna autumnalis* | 22.557 | 206.877 |
|  |  |  | *Dendrocygna autumnalis domestic duck** | 55.659 | 1369.002 |
|  |  |  | *Dendrocygna autumnalis domestic goose** | 75.610 | 1461.830 |
|  |  |  | *Mergus serrator* | 19.774 | 459.849 |
|  |  |  | *Somateria mollissima* | 31.643 | 729.628 |
|  |  |  | *Tadorna ferruginea* | 41.681 | 404.667 |
|  |  |  | *Tadorna tadorna* | 20.509 | 404.667 |
|  |  | **Charadriiformes** | |  |  |
|  |  |  | *Aethia cristatella* | 12.529 | 127.200 |
|  |  |  | *Aethia psittacula* | 10.934 | 119.500 |
|  |  |  | *Aethia pusilla* | 5.135 | 43.505 |
|  |  |  | *Alca torda* | 12.598 | 88.500 |
|  |  |  | *Alle alle* | 7.716 | 62.467 |
|  |  |  | *Anous minutus* | 5.076 | 53.938 |
|  |  |  | *Anous stolidus* | 4.893 | 95.000 |
|  |  |  | *Arenaria interpres* | 3.730 | 47.824 |
|  |  |  | *Burhinus oedicnemus* | 11.331 | 206.012 |
|  |  |  | *Calidris alba* | 2.533 | 29.798 |
|  |  |  | *Calidris alpina* | 2.031 | 14.715 |
|  |  |  | *Calidris bairdii* | 2.119 | 17.658 |
|  |  |  | *Calidris fuscicollis* | 2.151 | 13.612 |
|  |  |  | *Calidris melanotos* | 2.340 | 22.073 |
|  |  |  | *Calidris pusilla* | 1.444 | 9.565 |
|  |  |  | *Catharacta maccormicki* | 43.109 | 547.501 |
|  |  |  | *Catharacta skua* | 58.371 | 687.187 |
|  |  |  | *Cepphus columba* | 16.083 | 183.857 |
|  |  |  | *Cepphus grylle* | 14.553 | 158.188 |
|  |  |  | *Cerorhinca monocerata* | 9.832 | 150.210 |
|  |  |  | *Charadrius dubius* | 1.369 | 17.042 |
|  |  |  | *Charadrius hiaticula* | 2.328 | 22.815 |
|  |  |  | *Charadrius melodus* | 1.645 | 21.650 |
|  |  |  | *Chlidonias niger* | 5.749 | 31.500 |
|  |  |  | *Coenocorypha aucklandica aucklandica** | 2.791 | 39.878 |
|  |  |  | *Coenocorypha pusilla* | 2.153 | 29.099 |
|  |  |  | *Creagrus furcatus* | 17.452 | 326.200 |
|  |  |  | *Eudromias morinellus* | 4.818 | 40.835 |
|  |  |  | *Fratercula arctica* | 12.030 | 155.292 |
|  |  |  | *Gallinago gallinago* | 3.929 | 43.670 |
|  |  |  | *Gelochelidon nilotica** | 6.162 | 91.970 |
|  |  |  | *Gygis alba* | 2.400 | 50.000 |
|  |  |  | *Haematopus moquini* | 13.075 | 251.450 |
|  |  |  | *Haematopus ostralegus* | 13.886 | 171.432 |
|  |  |  | *Himantopus himantopus* | 5.644 | 64.500 |
|  |  |  | *Himantopus novaezelandiae* | 4.193 | 65.000 |
|  |  |  | *Hydroprogne caspia** | 28.215 | 270.000 |
|  |  |  | *Ichthyaetus audouinii** | 22.950 | 255.000 |
|  |  |  | *Larus argentatus* | 38.673 | 420.040 |
|  |  |  | *Larus californicus* | 24.000 | 300.000 |
|  |  |  | *Larus canus* | 19.130 | 147.152 |
|  |  |  | *Larus fuscus* | 25.550 | 321.723 |
|  |  |  | *Larus glaucescens* | 29.627 | 325.573 |
|  |  |  | *Larus marinus* | 54.713 | 616.198 |
|  |  |  | *Larus minutus* | 7.508 | 55.000 |
|  |  |  | *Larus occidentalis* | 28.644 | 365.546 |
|  |  |  | *Larus ridibundus* | 16.740 | 135.000 |
|  |  |  | *Limosa limosa* | 8.064 | 103.205 |
|  |  |  | *Numenius arquata* | 18.376 | 260.546 |
|  |  |  | *Pedionomus torquatus* | 1.510 | 18.762 |
|  |  |  | *Philomachus pugnax* | 5.441 | 60.700 |
|  |  |  | *Pluvialis apricaria* | 6.537 | 95.508 |
|  |  |  | *Pluvianus aegyptius* | 1.951 | 28.695 |
|  |  |  | *Ptychoramphus aleuticus* | 5.986 | 73.118 |
|  |  |  | *Recurvirostra avosetta* | 9.806 | 104.400 |
|  |  |  | *Rissa tridactyla* | 18.099 | 188.614 |
|  |  |  | *Rynchops niger* | 15.949 | 139.900 |
|  |  |  | *Stercorarius longicaudus* | 20.095 | 121.420 |
|  |  |  | *Stercorarius lonnbergi** | 56.940 | 780.000 |
|  |  |  | *Sterna albifrons* | 2.053 | 16.555 |
|  |  |  | *Sterna anaethetus* | 3.359 | 56.170 |
|  |  |  | *Sterna dougallii* | 6.562 | 50.083 |
|  |  |  | *Sterna forsteri* | 5.472 | 64.379 |
|  |  |  | *Sterna fuscata* | 5.508 | 79.637 |
|  |  |  | *Sterna hirundo* | 7.718 | 58.228 |
|  |  |  | *Sterna lunata* | 5.471 | 51.135 |
|  |  |  | *Sterna paradisaea* | 7.425 | 52.904 |
|  |  |  | *Sterna sumatrana* | 7.200 | 50.000 |
|  |  |  | *Sterna vittata* | 7.182 | 66.500 |
|  |  |  | *Thalasseus bergii** | 7.623 | 138.910 |
|  |  |  | *Thalasseus sandvicensis** | 10.541 | 98.120 |
|  |  |  | *Tringa totanus* | 3.528 | 50.399 |
|  |  |  | *Turnix suscitator* | 1.504 | 16.922 |
|  |  |  | *Turnix sylvaticus dussumier** | 0.803 | 9.675 |
|  |  |  | *Turnix sylvaticus lepurana** | 1.913 | 22.500 |
|  |  |  | *Uria aalge* | 16.796 | 224.021 |
|  |  |  | *Uria lomvia* | 10.908 | 103.130 |
|  |  |  | *Vanellus vanellus* | 6.223 | 101.107 |
|  |  | **Galliformes** | |  |  |
|  |  |  | *Alectoris graeca saxatilis** | 9.271 | 264.873 |
|  |  |  | *Alectura lathami* | 13.646 | 670.374 |
|  |  |  | *Bonasa bonasia* | 8.297 | 145.570 |
|  |  |  | *Bonasa umbellus* | 8.617 | 219.724 |
|  |  |  | *Callipepla californica* | 2.439 | 62.540 |
|  |  |  | *Coturnix chinensis* | 1.466 | 25.500 |
|  |  |  | *Coturnix coturnix* | 3.193 | 48.554 |
|  |  |  | *Coturnix delegorguei* | 0.815 | 10.800 |
|  |  |  | *Coturnix japonica* | 3.517 | 55.195 |
|  |  |  | *Cyrtonyx montezumae* | 5.445 | 68.058 |
|  |  |  | *Dendragapus canadensis* | 9.826 | 247.317 |
|  |  |  | *Dendragapus obscurus* | 12.140 | 314.575 |
|  |  |  | *Gallus gallus* | 5.886 | 235.443 |
|  |  |  | *Gallus gallus Domestic chicken** | 33.663 | 1309.099 |
|  |  |  | *Lagopus lagopus* | 14.383 | 244.311 |
|  |  |  | *Lagopus muta* | 12.596 | 226.950 |
|  |  |  | *Leipoa ocellata* | 4.110 | 587.136 |
|  |  |  | *Macrocephalon maleo* | 2.706 | 109.171 |
|  |  |  | *Meleagris gallopavo* | 37.965 | 2372.822 |
|  |  |  | *Meleagris gallopavo Domestic turkey** | 75.403 | 1653.802 |
|  |  |  | *Pavo cristatus* | 23.610 | 1184.265 |
|  |  |  | *Perdix perdix* | 7.598 | 139.474 |
|  |  |  | *Phasianus colchicus* | 12.787 | 488.776 |
|  |  |  | *Tetrao tetrix* | 16.468 | 429.481 |
|  |  |  | *Tetrao urogallus* | 36.941 | 1024.688 |
|  |  |  | *Tetrastes bonasia** | 3.975 | 150.000 |
|  |  |  | *Tympanuchus phasianellus* | 11.160 | 348.750 |
|  |  | **Gruiformes** | |  |  |
|  |  |  | *Anthropoides paradiseus** | 61.897 | 1535.897 |
|  |  |  | *Anthropoides virgo** | 49.026 | 860.102 |
|  |  |  | *Balearica regulorum gibbericeps** | 58.110 | 1320.687 |
|  |  |  | *Crex crex* | 4.972 | 67.000 |
|  |  |  | *Fulica americana* | 7.768 | 189.458 |
|  |  |  | *Fulica atra* | 21.750 | 250.000 |
|  |  |  | *Grus americana* | 113.925 | 2324.998 |
|  |  |  | *Grus antigone* | 91.367 | 2610.473 |
|  |  |  | *Grus canadensis pratensis** | 60.250 | 1882.807 |
|  |  |  | *Grus canadensis tabida** | 49.948 | 1314.433 |
|  |  |  | *Grus grus* | 65.764 | 1753.865 |
|  |  |  | *Grus japonensis* | 93.884 | 2761.303 |
|  |  |  | *Grus leucogeranus* | 97.948 | 1958.958 |
|  |  |  | *Grus monacha* | 59.630 | 1325.102 |
|  |  |  | *Grus rubicunda* | 92.626 | 1781.272 |
|  |  |  | *Grus vipio* | 75.696 | 1940.932 |
|  |  |  | *Lophotis ruficrista** | 11.683 | 243.389 |
|  |  |  | *Otis tarda* | 51.466 | 1949.761 |
|  |  |  | *Porzana carolina* | 2.476 | 36.725 |
|  |  |  | *Porzana porzana* | 3.205 | 30.902 |
|  |  |  | *Psophia spp.** | 16.319 | 253.000 |
|  |  |  | *Rallus aquaticus* | 3.300 | 51.927 |
|  |  |  | *Rallus elegans* | 6.353 | 165.000 |
|  |  |  | *Rallus limicola limicola** | 1.951 | 19.605 |
|  |  | **Passeriformes** | |  |  |
|  |  |  | *Picathartes gymnocephalus* | 11.239 | 92.500 |
|  |  |  | *Picathartes oreas* | 7.811 | 82.221 |
|  |  | **Procellariiformes** | |  |  |
|  |  |  | *Calonectris diomedea* | 13.551 | 371.250 |
|  |  |  | *Daption capense* | 19.735 | 268.500 |
|  |  |  | *Daption capense capense** | 33.465 | 345.000 |
|  |  |  | *Diomedea amsterdamensis* | 47.473 | 3651.792 |
|  |  |  | *Diomedea epomophora* | 72.840 | 4046.674 |
|  |  |  | *Diomedea exulans* | 95.685 | 4359.371 |
|  |  |  | *Fulmarus glacialoides* | 36.300 | 600.000 |
|  |  |  | *Halobaena caerulea* | 7.534 | 98.628 |
|  |  |  | *Macronectes giganteus* | 103.742 | 2207.277 |
|  |  |  | *Macronectes halli* | 83.877 | 2096.913 |
|  |  |  | *Oceanites oceanicus* | 1.728 | 22.441 |
|  |  |  | *Oceanodroma castro* | 2.340 | 40.000 |
|  |  |  | *Oceanodroma furcata* | 2.392 | 39.750 |
|  |  |  | *Oceanodroma homochroa* | 1.260 | 24.000 |
|  |  |  | *Oceanodroma leucorhoa* | 1.697 | 37.000 |
|  |  |  | *Pachyptila desolata* | 8.066 | 108.250 |
|  |  |  | *Pachyptila salvini* | 4.937 | 64.121 |
|  |  |  | *Pachyptila turtur* | 5.438 | 75.000 |
|  |  |  | *Pagodroma nivea* | 17.751 | 194.000 |
|  |  |  | *Pelecanoides georgicus* | 5.940 | 72.000 |
|  |  |  | *Pelecanoides urinatrix* | 5.063 | 78.500 |
|  |  |  | *Phoebastria immutabilis* | 23.544 | 1471.518 |
|  |  |  | *Phoebastria nigripes* | 32.373 | 1471.518 |
|  |  |  | *Phoebetria palpebrata* | 53.151 | 1236.075 |
|  |  |  | *Procellaria aequinoctialis* | 26.686 | 620.613 |
|  |  |  | *Procellaria cinerea* | 13.687 | 526.435 |
|  |  |  | *Pterodroma hypoleuca* | 5.688 | 125.000 |
|  |  |  | *Pterodroma macroptera* | 10.583 | 440.950 |
|  |  |  | *Pterodroma phaeopygia* | 9.307 | 169.225 |
|  |  |  | *Puffinus griseus* | 18.368 | 395.000 |
|  |  |  | *Puffinus pacificus* | 12.030 | 278.174 |
|  |  |  | *Thalassarche chrysostoma* | 81.102 | 1752.136 |
|  |  |  | *Thalassarche melanophris** | 80.399 | 1875.957 |
|  |  |  | *Thalassoica antarctica* | 19.198 | 270.391 |
|  |  | **Struthioniformes** | |  |  |
|  |  |  | *Apteryx australis* | 7.800 | 500.000 |
|  |  | **Tinamiformes** | |  |  |
|  |  |  | *Eudromia elegans* | 2.975 | 138.350 |
|  |  |  | *Nothura darwinii* | 4.132 | 87.923 |
|  |  |  | *Nothura maculosa* | 2.995 | 80.933 |
|  |  |  | *Rhynchotus rufescens* | 6.458 | 315.000 |
|  |  |  |  |  |  |
| **Mammals** | | |  |  |  |
|  | **Eutherians** | | |  |  |
|  |  | **Artiodactyla** | |  |  |
|  |  |  | *Aepyceros melampus* | 59.795 | 13905.843 |
|  |  |  | *Alces alces* | 464.852 | 119192.939 |
|  |  |  | *Ammotragus lervia* | 144.087 | 27186.291 |
|  |  |  | *Antidorcas marsupialis* | 129.527 | 9454.502 |
|  |  |  | *Antilocapra americana* | 126.105 | 15193.421 |
|  |  |  | *Bos taurus (Ayrshire)** | 653.354 | 204173.090 |
|  |  |  | *Bos taurus (Guernsey)** | 593.757 | 197919.139 |
|  |  |  | *Bos taurus (Holstein)** | 753.822 | 243168.311 |
|  |  |  | *Capra hircus* | 90.046 | 21962.403 |
|  |  |  | *Capra ibex* | 96.568 | 19313.671 |
|  |  |  | *Cervus elaphus canadensis** | 472.357 | 78726.200 |
|  |  |  | *Connochaetes taurinus* | 156.864 | 60332.228 |
|  |  |  | *Damaliscus pygargus* | 136.998 | 24463.983 |
|  |  |  | *Madoqua kirkii* | 24.177 | 1949.761 |
|  |  |  | *Odocoileus hemionus columbianus** | 99.684 | 20343.733 |
|  |  |  | *Odocoileus hemionus hemionus** | 205.733 | 33182.726 |
|  |  |  | *Odocoileus hemionus sitkensis** | 161.701 | 27407.018 |
|  |  |  | *Odocoileus virginianus* | 218.116 | 31159.389 |
|  |  |  | *Ovibos moschatus* | 389.143 | 94912.896 |
|  |  |  | *Ovis canadensis* | 80.400 | 25935.501 |
|  |  |  | *Potamochoerus porcus* | 171.903 | 17363.910 |
|  |  |  | *Pudu puda* | 43.656 | 2347.071 |
|  |  |  | *Rangifer tarandus* | 164.777 | 35058.911 |
|  |  |  | *Redunca fulvorufula* | 105.033 | 11293.899 |
|  |  |  | *Saiga tatarica* | 69.824 | 10742.080 |
|  |  |  | *Sus scrofa* | 324.470 | 54078.278 |
|  |  |  | *Sus scrofa (Duroc-Jersey)** | 1147.784 | 88291.066 |
|  |  |  | *Syncerus caffer* | 494.504 | 190193.671 |
|  |  |  | *Taurotragus oryx* | 366.960 | 193136.707 |
|  |  |  | *Tragelaphus imberbis* | 169.500 | 17842.153 |
|  |  |  | *Tragelaphus scriptus* | 46.132 | 10484.564 |
|  |  |  | *Vicugna vicugna* | 90.167 | 15818.816 |
|  |  | **Carnivora** | |  |  |
|  |  |  | *Abrocoma cinerea* | 1.021 | 55.182 |
|  |  |  | *Agouti paca* | 41.276 | 2501.580 |
|  |  |  | *Ailuropoda melanoleuca* | 115.367 | 41202.497 |
|  |  |  | *Ailurus fulgens* | 19.799 | 2199.919 |
|  |  |  | *Atherurus africanus* | 10.418 | 882.911 |
|  |  |  | *Bassariscus astutus* | 8.305 | 386.273 |
|  |  |  | *Canis familiaris (beagle)** | 59.229 | 3678.794 |
|  |  |  | *Canis familiaris (shepard)** | 249.014 | 7615.104 |
|  |  |  | *Canis latrans* | 74.054 | 4046.674 |
|  |  |  | *Canis lupus* | 185.577 | 10484.564 |
|  |  |  | *Canis mesomelas* | 38.754 | 2600.908 |
|  |  |  | *Capromys pilorides* | 18.983 | 2372.822 |
|  |  |  | *Caracal caracal* | 26.240 | 2733.344 |
|  |  |  | *Cavia aperea* | 2.223 | 209.691 |
|  |  |  | *Cavia porcellus* | 3.829 | 361.258 |
|  |  |  | *Cerdocyon thous* | 43.786 | 2637.696 |
|  |  |  | *Chrysocyon brachyurus* | 98.150 | 8461.227 |
|  |  |  | *Coendou prehensilis* | 18.582 | 1659.136 |
|  |  |  | *Crocuta crocuta* | 113.307 | 20233.369 |
|  |  |  | *Cryptoprocta ferox* | 11.519 | 3031.327 |
|  |  |  | *Ctenodactylus vali* | 1.728 | 56.286 |
|  |  |  | *Eira barbara* | 26.029 | 1637.064 |
|  |  |  | *Erethizon dorsatum* | 24.238 | 3068.115 |
|  |  |  | *Felis chaus* | 28.226 | 1710.639 |
|  |  |  | *Felis margarita* | 20.638 | 809.335 |
|  |  |  | *Felis silvestris* | 21.450 | 1898.258 |
|  |  |  | *Galea musteloides* | 2.217 | 122.504 |
|  |  |  | *Galidia elegans* | 2.703 | 290.625 |
|  |  |  | *Genetta genetta* | 4.341 | 735.759 |
|  |  |  | *Geocapromys ingrahami* | 3.339 | 269.288 |
|  |  |  | *Gulo gulo* | 67.624 | 3715.582 |
|  |  |  | *Hoplomys gymnurus* | 2.417 | 107.421 |
|  |  |  | *Hydrochaeris hydrochaeris* | 77.512 | 18026.093 |
|  |  |  | *Ictonyx striatus* | 12.085 | 334.770 |
|  |  |  | *Lynx lynx* | 67.086 | 6916.133 |
|  |  |  | *Lynx rufus* | 21.163 | 3112.260 |
|  |  |  | *Massoutiera mzabi* | 1.363 | 54.078 |
|  |  |  | *Meles meles* | 36.485 | 1861.470 |
|  |  |  | *Mephitis mephitis* | 10.521 | 735.759 |
|  |  |  | *Microcavia australis* | 1.563 | 95.281 |
|  |  |  | *Mustela nivalis* | 1.245 | 22.809 |
|  |  |  | *Mustela putorius* | 10.655 | 324.838 |
|  |  |  | *Myoprocta pratti** | 6.209 | 271.127 |
|  |  |  | *Napaeozapus insignis* | 0.245 | 8.829 |
|  |  |  | *Neovison vison** | 8.768 | 329.620 |
|  |  |  | *Octodon degus* | 2.400 | 74.312 |
|  |  |  | *Panthera leo* | 194.424 | 55549.796 |
|  |  |  | *Panthera onca* | 164.221 | 22808.525 |
|  |  |  | *Panthera pardus* | 113.344 | 14347.298 |
|  |  |  | *Paradoxurus hermaphroditus* | 8.962 | 1030.062 |
|  |  |  | *Pectinator spekei* | 1.000 | 90.130 |
|  |  |  | *Plagiodontia aedium* | 4.298 | 467.207 |
|  |  |  | *Poecilogale albinucha* | 2.580 | 101.167 |
|  |  |  | *Potos flavus* | 10.619 | 1041.099 |
|  |  |  | *Procyon lotor* | 29.662 | 1938.725 |
|  |  |  | *Proechimys guairae* | 2.390 | 112.203 |
|  |  |  | *Proechimys semispinosus* | 2.169 | 165.546 |
|  |  |  | *Profelis aurata* | 55.734 | 5518.192 |
|  |  |  | *Puma concolor* | 96.495 | 15818.816 |
|  |  |  | *Spilogale putorius* | 6.052 | 194.608 |
|  |  |  | *Suricata suricatta* | 3.554 | 220.728 |
|  |  |  | *Urocyon cinereoargenteus* | 22.893 | 1802.609 |
|  |  |  | *Ursus americanus* | 73.719 | 25420.469 |
|  |  |  | *Ursus maritimus* | 150.536 | 68425.576 |
|  |  |  | *Vulpes velox* | 26.073 | 827.729 |
|  |  |  | *Vulpes vulpes* | 35.813 | 2023.337 |
|  |  |  | *Zapus hudsonius* | 0.241 | 7.358 |
|  |  | **Cetacea** | |  |  |
|  |  |  | *Phocoena phocoena* | 194.240 | 22072.766 |
|  |  | **Chiroptera** | |  |  |
|  |  |  | *Antrozous pallidus* | 0.320 | 5.886 |
|  |  |  | *Carollia perspicillata* | 0.255 | 7.358 |
|  |  |  | *Desmodus rotundus* | 0.270 | 9.197 |
|  |  |  | *Eptesicus fuscus* | 0.388 | 6.254 |
|  |  |  | *Eptesicus serotinus* | 0.412 | 8.461 |
|  |  |  | *Miniopterus schreibersi* | 0.289 | 6.254 |
|  |  |  | *Myotis grisescens* | 0.284 | 2.943 |
|  |  |  | *Myotis lucifugus* | 0.341 | 2.943 |
|  |  |  | *Myotis myotis* | 0.842 | 7.725 |
|  |  |  | *Myotis thysanodes* | 0.318 | 2.943 |
|  |  |  | *Myotis velifer* | 0.526 | 4.047 |
|  |  |  | *Nyctalus lasiopterus* | 1.032 | 11.772 |
|  |  |  | *Nyctalus noctula* | 0.390 | 12.876 |
|  |  |  | *Nycticeius humeralis* | 0.069 | 4.415 |
|  |  |  | *Pipistrellus pipistrellus* | 0.123 | 1.839 |
|  |  |  | *Rousettus aegyptiacus** | 0.647 | 43.410 |
|  |  |  | *Tadarida brasiliensis* | 0.453 | 4.047 |
|  |  | **Edentata** | |  |  |
|  |  |  | *Chaetophractus villosus* | 34.268 | 1655.457 |
|  |  |  | *Choloepus didactylus* | 6.622 | 3310.915 |
|  |  |  | *Myrmecophaga tridactyla* | 90.645 | 10300.624 |
|  |  |  | *Tolypeutes matacus* | 15.981 | 441.455 |
|  |  | **Hyracoidea** | |  |  |
|  |  |  | *Dendrohyrax dorsalis* | 9.765 | 871.874 |
|  |  |  | *Procavia capensis* | 8.735 | 779.904 |
|  |  | **Insectivora** | |  |  |
|  |  |  | *Atelerix albiventris* | 4.319 | 110.732 |
|  |  |  | *Blarina brevicauda* | 0.761 | 4.047 |
|  |  |  | *Crocidura fuscomurina* | 0.147 | 1.472 |
|  |  |  | *Crocidura russula* | 0.430 | 2.943 |
|  |  |  | *Crocidura suaveolens* | 0.210 | 2.207 |
|  |  |  | *Cryptotis parva* | 0.197 | 1.472 |
|  |  |  | *Echinops telfairi* | 1.694 | 56.286 |
|  |  |  | *Microgale dobsoni* | 0.479 | 18.026 |
|  |  |  | *Microgale talazaci* | 0.471 | 15.819 |
|  |  |  | *Setifer setosus* | 4.008 | 95.649 |
|  |  |  | *Sorex cinereus* | 0.318 | 1.472 |
|  |  |  | *Sorex vagrans* | 0.292 | 1.839 |
|  |  |  | *Suncus etruscus* | 0.128 | 0.736 |
|  |  |  | *Suncus murinus* | 0.615 | 9.565 |
|  |  |  | *Tenrec ecaudatus* | 16.862 | 378.916 |
|  |  | **Lagomorpha** | |  |  |
|  |  |  | *Lepus americanus* | 17.789 | 577.571 |
|  |  |  | *Lepus californicus* | 18.210 | 809.335 |
|  |  |  | *Lepus europaeus* | 28.317 | 1482.554 |
|  |  |  | *Lepus othus* | 50.199 | 1695.924 |
|  |  |  | *Ochotona princeps* | 2.213 | 58.861 |
|  |  |  | *Ochotona rufescens* | 3.650 | 58.125 |
|  |  |  | *Oryctolagus cuniculus* | 20.130 | 882.911 |
|  |  |  | *Sylvilagus aquaticus* | 11.127 | 783.583 |
|  |  |  | *Sylvilagus floridanus* | 10.012 | 412.025 |
|  |  | **Macroscelidea** | |  |  |
|  |  |  | *Elephantulus rufescens* | 1.009 | 20.969 |
|  |  | **Perissodactyla** | |  |  |
|  |  |  | *Diceros bicornis* | 1311.122 | 397309.796 |
|  |  |  | *Equus burchellii* | 424.974 | 111835.350 |
|  |  | **Pholidota** | |  |  |
|  |  |  | *Manis pentadactyla* | 11.582 | 728.401 |
|  |  | **Pinnepedia** | |  |  |
|  |  |  | *Arctocephalus gazella* | 18.181 | 12986.144 |
|  |  |  | *Callorhinus ursinus* | 11.331 | 16186.695 |
|  |  |  | *Mirounga leonina* | 123.607 | 154509.365 |
|  |  |  | *Odobenus rosmarus* | 230.440 | 256044.091 |
|  |  |  | *Pagophilus groenlandicus** | 55.623 | 46352.810 |
|  |  |  | *Phoca vitulina* | 32.521 | 25015.802 |
|  |  | **Primates** | |  |  |
|  |  |  | *Arctocebus calabarensis* | 0.965 | 158.188 |
|  |  |  | *Callimico goeldii* | 2.066 | 213.002 |
|  |  |  | *Callithrix argentata* | 3.408 | 577.571 |
|  |  |  | *Callithrix jacchus* | 1.221 | 71.001 |
|  |  |  | *Galago senegalensis* | 1.424 | 84.244 |
|  |  |  | *Gorilla gorilla* | 25.016 | 31269.752 |
|  |  |  | *Homo sapiens* | 11.276 | 22551.010 |
|  |  |  | *Hylobates lar* | 5.003 | 2501.580 |
|  |  |  | *Leontopithecus rosalia* | 1.955 | 253.837 |
|  |  |  | *Macaca mulatta* | 3.527 | 2939.357 |
|  |  |  | *Macaca nemestrina* | 2.787 | 1857.791 |
|  |  |  | *Microcebus murinus* | 0.932 | 25.752 |
|  |  |  | *Nasalis larvatus* | 6.536 | 3630.970 |
|  |  |  | *Pan troglodytes* | 11.459 | 16370.635 |
|  |  |  | *Papio sp.** | 7.709 | 2965.108 |
|  |  |  | *Perodicticus potto* | 6.208 | 496.637 |
|  |  |  | *Pongo pygmaeus* | 12.184 | 13537.963 |
|  |  |  | *Saguinus nigricollis* | 1.395 | 176.582 |
|  |  |  | *Saguinus oedipus* | 1.914 | 187.619 |
|  |  |  | *Saimiri sciureus* | 2.373 | 189.826 |
|  |  | **Proboscidea** | |  |  |
|  |  |  | *Loxodonta africana* | 305.708 | 1019026.052 |
|  |  | **Rodentia** | |  |  |
|  |  |  | *Acomys cahirinus* | 0.292 | 19.865 |
|  |  |  | *Aethomys chrysophilus* | 0.880 | 29.430 |
|  |  |  | *Aethomys hindei* | 1.581 | 70.265 |
|  |  |  | *Aethomys kaiseri* | 2.469 | 71.369 |
|  |  |  | *Akodon molinae* | 0.645 | 18.026 |
|  |  |  | *Ammospermophilus leucurus* | 1.609 | 56.653 |
|  |  |  | *Aplodontia rufa* | 8.582 | 297.982 |
|  |  |  | *Apodemus argenteus* | 0.247 | 4.415 |
|  |  |  | *Arborimus longicaudus* | 0.487 | 11.772 |
|  |  |  | *Arvicola terrestris* | 2.635 | 45.985 |
|  |  |  | *Baiomys taylori* | 0.141 | 3.311 |
|  |  |  | *Calomys callosus* | 0.572 | 11.404 |
|  |  |  | *Calomys lepidus* | 0.162 | 9.933 |
|  |  |  | *Cannomys badius* | 2.006 | 138.323 |
|  |  |  | *Castor fiber* | 95.649 | 9196.986 |
|  |  |  | *Chaetodipus californicus* | 0.536 | 10.669 |
|  |  |  | *Chiropodomys gliroides* | 0.314 | 8.093 |
|  |  |  | *Clethrionomys gapperi* | 0.631 | 9.933 |
|  |  |  | *Clethrionomys glareolus* | 0.744 | 9.197 |
|  |  |  | *Clethrionomys rutilus* | 0.428 | 9.197 |
|  |  |  | *Cricetomys gambianus* | 16.996 | 404.667 |
|  |  |  | *Cynomys ludovicianus* | 4.083 | 367.879 |
|  |  |  | *Cynomys mexicanus* | 9.359 | 378.916 |
|  |  |  | *Desmodillus auricularis* | 0.705 | 21.705 |
|  |  |  | *Dicrostonyx groenlandicus* | 1.154 | 28.695 |
|  |  |  | *Dicrostonyx unalascensis* | 0.433 | 29.430 |
|  |  |  | *Dipodomys heermanni* | 0.585 | 22.073 |
|  |  |  | *Dipodomys merriami* | 0.514 | 13.979 |
|  |  |  | *Dipodomys nitratoides* | 0.662 | 14.715 |
|  |  |  | *Dipodomys stephensi* | 1.363 | 18.026 |
|  |  |  | *Eliomys quercinus* | 1.083 | 26.487 |
|  |  |  | *Funambulus pennantii* | 0.649 | 31.638 |
|  |  |  | *Gerbillus pyramidum* | 0.581 | 17.658 |
|  |  |  | *Glaucomys sabrinus* | 0.450 | 51.135 |
|  |  |  | *Grammomys dolichurus* | 0.613 | 25.016 |
|  |  |  | *Heterocephalus glaber* | 0.059 | 12.876 |
|  |  |  | *Heteromys desmarestianus* | 2.258 | 22.809 |
|  |  |  | *Hydromys chrysogaster* | 4.024 | 240.961 |
|  |  |  | *Lemmus lemmus* | 0.397 | 18.026 |
|  |  |  | *Lemmus sibiricus* | 0.288 | 24.648 |
|  |  |  | *Lemniscomys striatus* | 0.548 | 20.601 |
|  |  |  | *Leopoldamys sabanus* | 1.997 | 121.032 |
|  |  |  | *Liomys pictus* | 0.774 | 18.394 |
|  |  |  | *Liomys salvini* | 1.081 | 13.979 |
|  |  |  | *Lophuromys sikapusi* | 1.058 | 25.016 |
|  |  |  | *Mastomys natalensis* | 0.544 | 10.301 |
|  |  |  | *Maxomys surifer* | 0.450 | 58.493 |
|  |  |  | *Meriones crassus* | 1.064 | 29.062 |
|  |  |  | *Meriones hurrianae* | 0.798 | 25.752 |
|  |  |  | *Meriones unguiculatus* | 0.691 | 21.337 |
|  |  |  | *Mesembriomys gouldii* | 12.270 | 375.237 |
|  |  |  | *Mesocricetus auratus* | 2.281 | 39.731 |
|  |  |  | *Microtus abbreviatus* | 0.304 | 19.865 |
|  |  |  | *Microtus arvalis* | 0.495 | 8.829 |
|  |  |  | *Microtus californicus* | 0.844 | 13.244 |
|  |  |  | *Microtus miurus* | 0.175 | 12.508 |
|  |  |  | *Microtus ochrogaster* | 0.858 | 12.876 |
|  |  |  | *Microtus oeconomus* | 0.764 | 9.565 |
|  |  |  | *Microtus oregoni* | 0.702 | 9.933 |
|  |  |  | *Microtus pennsylvanicus* | 0.540 | 13.612 |
|  |  |  | *Microtus pinetorum* | 0.607 | 11.036 |
|  |  |  | *Millardia meltada* | 1.121 | 23.544 |
|  |  |  | *Mus minutoides* | 0.184 | 2.207 |
|  |  |  | *Mus musculoides* | 0.076 | 2.575 |
|  |  |  | *Mus musculus* | 0.439 | 14.715 |
|  |  |  | *Neofiber alleni* | 3.801 | 89.027 |
|  |  |  | *Neotoma cinerea* | 3.626 | 82.037 |
|  |  |  | *Neotoma floridana* | 3.537 | 54.078 |
|  |  |  | *Neotoma micropus* | 2.440 | 86.820 |
|  |  |  | *Notomys alexis* | 0.396 | 12.140 |
|  |  |  | *Notomys cervinus* | 0.347 | 5.518 |
|  |  |  | *Notomys mitchellii* | 0.546 | 6.622 |
|  |  |  | *Ochrotomys nuttalli* | 0.435 | 7.358 |
|  |  |  | *Oligoryzomys eliurus* | 0.436 | 8.093 |
|  |  |  | *Ondatra zibethicus* | 9.649 | 298.718 |
|  |  |  | *Onychomys leucogaster* | 0.700 | 11.036 |
|  |  |  | *Onychomys torridus* | 0.159 | 8.829 |
|  |  |  | *Oryzomys palustris* | 0.664 | 21.705 |
|  |  |  | *Otomys irroratus* | 1.529 | 41.202 |
|  |  |  | *Ototylomys phyllotis* | 0.490 | 44.146 |
|  |  |  | *Paraxerus cepapi* | 2.358 | 81.301 |
|  |  |  | *Perognathus longimembris* | 0.225 | 2.943 |
|  |  |  | *Peromyscus californicus* | 0.685 | 16.922 |
|  |  |  | *Peromyscus eremicus* | 0.283 | 5.518 |
|  |  |  | *Peromyscus gossypinus* | 0.600 | 9.933 |
|  |  |  | *Peromyscus leucopus* | 0.386 | 8.461 |
|  |  |  | *Peromyscus maniculatus* | 0.352 | 6.622 |
|  |  |  | *Peromyscus maniculatus gracilis** | 0.376 | 7.358 |
|  |  |  | *Peromyscus maniculatus labecula** | 0.509 | 7.725 |
|  |  |  | *Peromyscus maniculatus nebrascensis** | 0.369 | 6.990 |
|  |  |  | *Peromyscus maniculatus nubiterrae** | 0.397 | 6.622 |
|  |  |  | *Peromyscus megalops* | 0.828 | 26.119 |
|  |  |  | *Peromyscus melanocarpus* | 0.645 | 22.809 |
|  |  |  | *Peromyscus mexicanus* | 0.849 | 23.912 |
|  |  |  | *Peromyscus oreas* | 0.332 | 6.622 |
|  |  |  | *Peromyscus polionotus* | 0.287 | 5.150 |
|  |  |  | *Peromyscus truei* | 0.478 | 9.933 |
|  |  |  | *Peromyscus yucatanicus* | 0.440 | 10.301 |
|  |  |  | *Phenacomys ungava* | 0.550 | 9.933 |
|  |  |  | *Podomys floridanus* | 0.450 | 8.461 |
|  |  |  | *Psammomys obesus* | 1.655 | 54.446 |
|  |  |  | *Pseudomys australis* | 0.501 | 18.026 |
|  |  |  | *Pseudomys gracilicaudatus* | 0.581 | 25.384 |
|  |  |  | *Pseudomys higginsi* | 0.755 | 22.809 |
|  |  |  | *Pseudomys novaehollandiae* | 0.216 | 5.518 |
|  |  |  | *Rattus exulans* | 0.823 | 23.176 |
|  |  |  | *Rattus fuscipes* | 0.734 | 40.099 |
|  |  |  | *Rattus lutreolus* | 1.162 | 41.938 |
|  |  |  | *Rattus rattus* | 1.066 | 51.503 |
|  |  |  | *Reithrodontomys humulis* | 0.153 | 2.207 |
|  |  |  | *Rhabdomys pumilio* | 0.611 | 16.922 |
|  |  |  | *Sciurus carolinensis* | 3.659 | 156.349 |
|  |  |  | *Sciurus niger* | 5.079 | 282.164 |
|  |  |  | *Sciurus vulgaris* | 2.318 | 110.364 |
|  |  |  | *Sigmodon hispidus* | 1.318 | 36.420 |
|  |  |  | *Sigmodon ochrognathus* | 0.874 | 35.684 |
|  |  |  | *Spermophilus armatus* | 7.632 | 159.660 |
|  |  |  | *Spermophilus beldingi* | 4.672 | 85.716 |
|  |  |  | *Spermophilus columbianus* | 8.310 | 157.085 |
|  |  |  | *Spermophilus franklinii* | 8.196 | 259.355 |
|  |  |  | *Spermophilus lateralis* | 3.236 | 78.726 |
|  |  |  | *Spermophilus richardsonii* | 7.095 | 126.918 |
|  |  |  | *Spermophilus tereticaudus* | 1.480 | 69.161 |
|  |  |  | *Spermophilus tridecemlineatus* | 1.519 | 64.379 |
|  |  |  | *Spermophilus undulatus* | 18.276 | 294.304 |
|  |  |  | *Sundamys muelleri* | 1.323 | 135.012 |
|  |  |  | *Tachyoryctes ruandae* | 1.346 | 76.887 |
|  |  |  | *Tamias amoenus* | 0.562 | 19.865 |
|  |  |  | *Tamias palmeri* | 0.829 | 26.487 |
|  |  |  | *Tamias panamintinus* | 0.777 | 18.762 |
|  |  |  | *Tamias quadrivittatus* | 0.657 | 27.591 |
|  |  |  | *Tamias townsendii* | 1.188 | 35.684 |
|  |  |  | *Tamiasciurus hudsonicus* | 1.518 | 70.265 |
|  |  |  | *Tatera brantsii* | 1.718 | 36.788 |
|  |  |  | *Tatera indica* | 0.618 | 25.752 |
|  |  |  | *Thomomys talpoides* | 2.205 | 35.684 |
|  |  |  | *Tylomys nudicaudus* | 3.535 | 112.939 |
|  |  |  | *Zelotomys woosnami* | 1.289 | 18.394 |
|  |  | **Scandentia** | |  |  |
|  |  |  | *Tupaia belangeri* | 2.583 | 73.576 |
|  |  | **Sirenia** | |  |  |
|  |  |  | *Trichechus manatus* | 164.221 | 68425.576 |
|  |  | **Tubulidentata** | |  |  |
|  |  |  | *Orycteropus afer* | 207.484 | 22072.766 |
|  |  |  |  |  |  |
|  | **Marsupialia** | | |  |  |
|  |  | **Marsupialia** | |  |  |
|  |  |  | *Acrobates pygmaeus* | 0.121 | 4.782 |
|  |  |  | *Antechinus flavipes* | 0.284 | 8.093 |
|  |  |  | *Bettongia lesueur* | 8.316 | 467.207 |
|  |  |  | *Didelphis virginiana* | 9.823 | 761.510 |
|  |  |  | *Isoodon macrourus* | 5.548 | 382.595 |
|  |  |  | *Isoodon obesulus* | 4.281 | 317.112 |
|  |  |  | *Macropus fuliginosus* | 51.650 | 4782.433 |
|  |  |  | *Macropus giganteus* | 60.700 | 9196.986 |
|  |  |  | *Macropus parma* | 13.170 | 1317.008 |
|  |  |  | *Macropus robustus* | 22.087 | 5812.495 |
|  |  |  | *Macropus rufus* | 70.909 | 9454.502 |
|  |  |  | *Perameles gunnii* | 4.477 | 315.273 |
|  |  |  | *Perameles nasuta* | 4.361 | 316.008 |
|  |  |  | *Petaurus breviceps* | 1.107 | 58.861 |
|  |  |  | *Petaurus norfolcensis* | 1.914 | 76.887 |
|  |  |  | *Phascolarctos cinereus* | 6.644 | 2372.822 |
|  |  |  | *Potorous tridactylus* | 6.059 | 496.637 |
|  |  |  | *Setonix brachyurus* | 9.297 | 1147.784 |
|  |  |  | *Thylogale billardierii* | 17.738 | 1809.967 |
|  |  |  | *Trichosurus vulpecula* | 9.852 | 478.243 |
|  |  |  | *Wallabia bicolor* | 25.266 | 3715.582 |
|  |  |  |  |  |  |
| **Reptiles** | | |  |  |  |
|  |  | **Chelonia** | |  |  |
|  |  |  | *Caretta caretta** | 12.100 | 57777.778 |
|  |  |  | *Chelonia mydas** | 10.520 | 50888.889 |
|  |  |  | *Chrysemys picta** | 0.120 | 103.704 |
|  |  |  | *Clemmys guttata** | 0.400 | 50.370 |
|  |  |  | *Geochelone elephantopus** | 31.500 | 59259.259 |
|  |  |  | *Geochelone sulcata** | 13.962 | 12568.237 |
|  |  |  | *Gopherus agassizi** | 1.680 | 829.630 |
|  |  | **Crocodilia** | |  |  |
|  |  |  | *Alligator mississippiensis** | 23.119 | 44548.020 |
|  |  |  | *Caiman crocodilus crocodilus** | 3.621 | 2829.896 |
|  |  |  | *Caiman latirostris** | 1.209 | 3437.630 |
|  |  |  | *Crocodylus porosus** | 2.750 | 28332.741 |
|  |  | **Lacertilia** | |  |  |
|  |  |  | *Anolis acutus* | 0.010 | 1.481 |
|  |  |  | *Aspidoscelis sexlineata** | 0.070 | 7.704 |
|  |  |  | *Aspidoscelis tigris* | 0.100 | 1.778 |
|  |  |  | *Callisaurus draconoides* | 0.088 | 5.037 |
|  |  |  | *Dipsosaurus dorsalis* | 0.100 | 17.778 |
|  |  |  | *Emoia atrocostata* | 0.050 | 5.037 |
|  |  |  | *Gymnophthalmus speciosus* | 0.003 | 0.593 |
|  |  |  | *Iguana iguana* | 0.210 | 444.444 |
|  |  |  | *Liolaemus signifer** | 0.010 | 6.519 |
|  |  |  | *Oligosoma zelandicum* | 0.010 | 1.185 |
|  |  |  | *Phrynosoma solare* | 0.250 | 14.815 |
|  |  |  | *Plestiodon fasciatus* | 0.030 | 6.519 |
|  |  |  | *Sauromalus ater* | 0.140 | 47.407 |
|  |  |  | *Sceloporus graciosus* | 0.020 | 1.778 |
|  |  |  | *Sceloporus magister* | 0.060 | 8.296 |
|  |  |  | *Sceloporus undulatus* | 0.050 | 4.444 |
|  |  |  | *Uta stansburiana* | 0.040 | 1.481 |
|  |  |  | *Xantusia vigilis* | 0.002 | 0.889 |
|  |  | **Rhynchocephalia** | |  |  |
|  |  |  | *Sphenodon punctatus* | 0.070 | 207.407 |
|  |  | **Serpentes** | |  |  |
|  |  |  | *Boa constrictor* | 2.400 | 23703.704 |
|  |  |  | *Bogertophis subocularis* | 0.060 | 355.556 |
|  |  |  | *Broghammerus reticulatus* | 10.100 | 17777.778 |
|  |  |  | *Carphophis vermis** | 0.035 | 2.074 |
|  |  |  | *Coluber constrictor* | 0.120 | 59.259 |
|  |  |  | *Crotalus viridis* | 0.100 | 77.037 |
|  |  |  | *Diadophis punctatus* | 0.010 | 14.815 |
|  |  |  | *Heterodon platirhinos* | 0.500 | 59.259 |
|  |  |  | *Nerodia erythrogaster* | 0.860 | 47.407 |
|  |  |  | *Python molurus* | 17.900 | 11851.852 |
|  |  |  | *Python sebae* | 9.600 | 2962.963 |
|  |  |  | *Spalerosophis diadema* | 0.070 | 148.148 |
|  |  |  | *Thamnophis sirtalis* | 0.100 | 59.259 |
|  |  | **Varanidae** | |  |  |
|  |  |  | *Varanus brevicauda* | 0.020 | 3.644 |
|  |  |  | *Varanus griseus* | 0.450 | 191.704 |
|  |  |  | *Varanus komodoensis* | 17.000 | 18814.815 |
|  |  |  | *Varanus niloticus* | 3.966 | 1926.139 |
|  |  |  | *Varanus salvator* | 5.817 | 5925.926 |
|  |  |  | *Varanus varius* | 1.280 | 1392.593 |
|  |  |  |  |  |  |
| **Fishes** | |  |  |  |  |
|  |  | **Acipenseriformes** | |  |  |
|  |  |  | *Acipenser fulvescens* | 2.192 | 10666.667 |
|  |  |  | *Acipenser transmontanus** | 8.565 | 39508.148 |
|  |  | **Aulopiformes** | |  |  |
|  |  |  | *Saurida tumbil** | 0.818 | 696.296 |
|  |  | **Beloniformes** | |  |  |
|  |  |  | *Cololabis saira* | 0.097 | 56.000 |
|  |  | **Clupeiformes** | |  |  |
|  |  |  | *Brevoortia tyrannus** | 0.191 | 148.741 |
|  |  |  | *Cetengraulis mysticetus** | 0.136 | 16.593 |
|  |  |  | *Clupea harengus** | 0.079 | 56.770 |
|  |  |  | *Clupea pallasii pallasii** | 0.122 | 108.741 |
|  |  |  | *Engraulis anchoita** | 0.057 | 38.815 |
|  |  |  | *Engraulis encrasicolus** | 0.033 | 7.111 |
|  |  |  | *Engraulis japonicus** | 0.079 | 10.667 |
|  |  |  | *Engraulis mordax** | 0.012 | 6.222 |
|  |  |  | *Engraulis ringens** | 0.056 | 9.037 |
|  |  |  | *Sardinella longiceps** | 0.044 | 19.111 |
|  |  |  | *Sardinops sagax** | 0.170 | 76.148 |
|  |  |  | *Sprattus sprattus** | 0.018 | 6.430 |
|  |  |  | *Stolothrissa tanganicae** | 0.019 | 1.778 |
|  |  | **Cypriniformes** | |  |  |
|  |  |  | *Abramis brama** | 0.452 | 472.000 |
|  |  |  | *Phoxinus phoxinus** | 0.006 | 2.430 |
|  |  | **Cyprinodontiformes** | |  |  |
|  |  |  | *Aphanius fasciatus** | 0.003 | 0.963 |
|  |  | **Esociformes** | |  |  |
|  |  |  | *Esox lucius* | 1.660 | 1792.296 |
|  |  | **Gadiformes** | |  |  |
|  |  |  | *Gadus morhua* | 2.453 | 3952.658 |
|  |  |  | *Melanogrammus aeglefinus* | 0.733 | 637.037 |
|  |  |  | *Merluccius angustimanus** | 0.109 | 75.556 |
|  |  |  | *Merluccius gayi gayi** | 0.852 | 1727.407 |
|  |  |  | *Merluccius merluccius** | 0.145 | 325.778 |
|  |  |  | *Merluccius productus* | 0.465 | 376.889 |
|  |  |  | *Pollachius virens* | 2.962 | 4762.963 |
|  |  |  | *Trisopterus esmarkii** | 0.029 | 13.778 |
|  |  |  | *Trisopterus minutus** | 0.094 | 32.207 |
|  |  | **Gasterosteiformes** | |  |  |
|  |  |  | *Gasterosteus aculeatus* | 0.001 | 0.533 |
|  |  |  | *Pungitius pungitius* | 0.001 | 0.119 |
|  |  | **Lamniformes** | |  |  |
|  |  |  | *Cetorhinus maximus** | 757.260 | 4094814.815 |
|  |  |  | *Lamna nasus** | 22.545 | 49422.222 |
|  |  | **Mugiliformes** | |  |  |
|  |  |  | *Mugil cephalus* | 1.172 | 725.926 |
|  |  | **Myctophiformes** | |  |  |
|  |  |  | *Benthosema glaciale* | 0.003 | 1.674 |
|  |  |  | *Myctophum punctatum* | 0.003 | 1.956 |
|  |  | **Osmeriformes** | |  |  |
|  |  |  | *Hypomesus olidus** | 0.030 | 4.444 |
|  |  |  | *Mallotus villosus* | 0.035 | 17.778 |
|  |  | **Perciformes** | |  |  |
|  |  |  | *Ammodytes marinus** | 0.035 | 9.511 |
|  |  |  | *Ammodytes tobianus** | 0.014 | 4.444 |
|  |  |  | *Apsilus dentatus* | 2.511 | 1259.259 |
|  |  |  | *Atractoscion nobilis ** | 4.348 | 8266.667 |
|  |  |  | *Callionymus lyra** | 0.011 | 5.156 |
|  |  |  | *Caranx ruber** | 0.728 | 737.778 |
|  |  |  | *Cephalopholis fulva* | 0.491 | 189.630 |
|  |  |  | *Copadichromis mloto** | 0.050 | 21.926 |
|  |  |  | *Cynoscion analis** | 0.214 | 434.370 |
|  |  |  | *Dicentrarchus labrax* | 1.169 | 2032.593 |
|  |  |  | *Epinephelus guttatus** | 0.549 | 557.037 |
|  |  |  | *Epinephelus striatus** | 1.414 | 3822.222 |
|  |  |  | *Eubleekeria splendens** | 0.081 | 18.963 |
|  |  |  | *Haemulon album** | 1.291 | 1570.370 |
|  |  |  | *Haemulon plumierii* | 0.580 | 402.963 |
|  |  |  | *Katsuwonus pelamis* | 11.836 | 6285.926 |
|  |  |  | *Lethrinops longipinnis** | 0.085 | 36.148 |
|  |  |  | *Lethrinops parvidens** | 0.078 | 39.111 |
|  |  |  | *Lethrinus enigmaticus** | 0.579 | 864.000 |
|  |  |  | *Lipophrys pholis** | 0.020 | 16.000 |
|  |  |  | *Lutjanus apodus** | 0.833 | 1125.926 |
|  |  |  | *Lutjanus buccanella** | 1.767 | 754.074 |
|  |  |  | *Lutjanus purpureus** | 1.363 | 3456.000 |
|  |  |  | *Mulloidichthys martinicus** | 0.214 | 130.370 |
|  |  |  | *Mycteroperca venenosa** | 1.826 | 2468.148 |
|  |  |  | *Mylochromis anaphyrmus** | 0.091 | 32.889 |
|  |  |  | *Nemadactylus macropterus** | 0.472 | 411.852 |
|  |  |  | *Nemipterus bipunctatus** | 0.183 | 80.000 |
|  |  |  | *Nemipterus hexodon** | 0.134 | 63.704 |
|  |  |  | *Nemipterus japonicus** | 0.142 | 75.556 |
|  |  |  | *Nemipterus marginatus** | 0.105 | 65.185 |
|  |  |  | *Nemipterus mesoprion** | 0.057 | 22.222 |
|  |  |  | *Nemipterus nematophorus** | 0.240 | 59.259 |
|  |  |  | *Nemipterus nemurus** | 0.082 | 72.593 |
|  |  |  | *Nemipterus peronii** | 0.139 | 72.148 |
|  |  |  | *Nemipterus sp.** | 0.223 | 120.296 |
|  |  |  | *Notothenia neglecta** | 0.286 | 645.333 |
|  |  |  | *Ocyurus chrysurus* | 1.096 | 1066.667 |
|  |  |  | *Oreochromis esculentus** | 0.256 | 222.222 |
|  |  |  | *Perca fluviatilis* | 0.103 | 158.222 |
|  |  |  | *Pomatoschistus minutus** | 0.004 | 1.185 |
|  |  |  | *Protonibea diacanthus** | 6.674 | 5155.556 |
|  |  |  | *Pseudotolithus elongatus** | 0.239 | 211.852 |
|  |  |  | *Pseudupeneus maculatus* | 0.307 | 106.667 |
|  |  |  | *Rastrelliger brachysoma** | 0.484 | 39.407 |
|  |  |  | *Rastrelliger kanagurta** | 0.730 | 46.519 |
|  |  |  | *Sander canadensis** | 0.097 | 182.222 |
|  |  |  | *Scomber japonicus* | 0.395 | 240.000 |
|  |  |  | *Thunnus alalunga** | 6.321 | 9956.543 |
|  |  |  | *Thunnus albacares* | 71.056 | 42953.333 |
|  |  |  | *Thunnus atlanticus** | 3.922 | 2891.852 |
|  |  |  | *Thunnus maccoyii** | 35.975 | 57973.333 |
|  |  |  | *Totoaba macdonaldi** | 6.512 | 5281.778 |
|  |  |  | *Trachurus japonicus** | 0.405 | 352.000 |
|  |  | **Pleuronectiformes** | |  |  |
|  |  |  | *Cynoglossus arel** | 0.049 | 50.370 |
|  |  |  | *Eopsetta jordani* | 0.848 | 1264.593 |
|  |  |  | *Hippoglossoides platessoides** | 0.235 | 612.185 |
|  |  |  | *Limanda ferruginea** | 0.483 | 350.519 |
|  |  |  | *Platichthys flesus** | 0.431 | 308.148 |
|  |  |  | *Pleuronectes platessa* | 0.250 | 642.963 |
|  |  |  | *Pseudopleuronectes americanus* | 0.672 | 408.889 |
|  |  |  | *Solea solea* | 0.247 | 142.815 |
|  |  | **Salmoniformes** | |  |  |
|  |  |  | *Coregonus artedi** | 0.098 | 75.556 |
|  |  |  | *Coregonus clupeaformis* | 0.567 | 982.815 |
|  |  |  | *Coregonus sardinella** | 0.268 | 162.963 |
|  |  |  | *Salmo trutta** | 0.149 | 118.519 |
|  |  |  | *Salvelinus alpinus* | 0.578 | 1481.481 |
|  |  | **Scorpaeniformes** | |  |  |
|  |  |  | *Cottus gobio** | 0.004 | 1.526 |
|  |  |  | *Sebastes alutus** | 0.170 | 319.901 |
|  |  | **Tetraodontiformes** | |  |  |
|  |  |  | *Balistes vetula* | 2.027 | 865.185 |
|  |  |  |  |  |  |
| **Non-avian dinosaurs** | | | |  |  |
|  |  | **Cerapoda** | |  |  |
|  |  |  | *Psittacosaurus luijatunensis (N=20) Femur* | 13.990 | 18690.000 |
|  |  |  | *Psittacosaurus mongoliensis (N=7)* | 15.950 | 12000.000 |
|  |  | **Sauropodomorpha** | |  |  |
|  |  |  | *Alamosaurus (N=9) Humerus A* | 2589.040 | 9481481.481 |
|  |  |  | *Alamosaurus (N=9) Humerus B* | 2986.300 | 9481481.481 |
|  |  |  | *Alamosaurus (N=9) Humerus C* | 3178.080 | 9481481.481 |
|  |  |  | *Apatosaurus BYU601-17328 (N=18) A* | 4421.400 | 9089000.000 |
|  |  |  | *Apatosaurus SMA0014 (N=20) B* | 4024.100 | 10103000.000 |
|  |  |  | *Camarasaurus CM3664 (N=9)* | 4188.800 | 7123500.000 |
|  |  |  | *cf. Mamenchisaurus (N=22)* | 3907.700 | 12537500.000 |
|  |  |  | *indet.diplo MfM.R.2625 (N=9) A* | 901.100 | 2376500.000 |
|  |  |  | *indet.diplo MfM.R.NW4 (N=16) B* | 2335.100 | 9231500.000 |
|  |  |  | *Massospondylus carinatus (N=9)* | 94.790 | 140000.000 |
|  |  |  | *Plateosaurus (N=12) A* | 819.200 | 793500.000 |
|  |  |  | *Plateosaurus (N=12) B* | 680.500 | 793500.000 |
|  |  | **Theropoda** | |  |  |
|  |  |  | *Albertosaurus sacrophagus (N=5)* | 334.250 | 614000.000 |
|  |  |  | *Archaeopterix (N=10) Femur A* | 1.870 | 464.050 |
|  |  |  | *Archaeopterix (N=10) Femur B* | 2.200 | 464.050 |
|  |  |  | *Gorgosaurus libratus (N=5)* | 394.520 | 622000.000 |
|  |  |  | *Tyrannosaurus rex (N=7)* | 2101.370 | 2780000.000 |

References

Birds:

Starck JM, Ricklefs RE (1998) Avian growth rate data set. In: Starck JM, Ricklefs RE, editors. Avian growth and development. Evolution within the altricial precocial spectrum. New York: Oxford University Press. pp. 381-415.

Mammals:

Zullinger EM, Ricklefs RE, Redford KH, Mace GM (1984) Fitting sigmoidal equations to mammalian growth curves. J Mammal 65: 607-636.

Non-avian dinosaurs:

Griebeler EM (2013) Body Temperatures in Dinosaurs: What Can Growth Curves Tell Us? PLoS ONE 8: e74317.

Reptiles:

Case TJ (1978) On the evolution and adaptive significance of postnatal-growth rates in terrestrial vertebrates. Q Rev Biol 53: 243-282.

Griebeler EM (2013) Body Temperatures in Dinosaurs: What Can Growth Curves Tell Us? PLoS ONE 8: e74317.

Ritz J, Griebeler EM, Huber R, Clauss M (2010) Body size development of captive and free-ranging African spurred tortoises (Geochelone sulcata): high plasticity in reptilian growth rates. The Herpetological Journal 20: 213-216.

Bjorndal KA, Bolten AB, Chaloupka MY (2000) Green turtle somatic growth model: evidence for density dependence. Ecol Appl 10: 269-282.

Fishes:

Pauly D (1980) On the interrelationships between natural mortality, growth parameters, and mean environmental temperature in 175 fish stocks. Journal du Conseil 39: 175-192.
