# Supplementary material for: Allometries of Maximum Growth Rate versus Body Mass at Maximum Growth Indicate That Non-Avian Dinosaurs Had Growth Rates Typical of Fast Growing Ectothermic Sauropsids
Source: PLoS One. 2014 Feb 25;9(2):e88834. doi: 10.1371/journal.pone.0088834 (PMC3934860; doi:10.1371/journal.pone.0088834)
Supplement: Table S2 — Ordinary linear least square (OLS) regression models of maximum absolute growth rate per day (AGR) on body mass at maximum growth (BMatMG, in gram) for different taxonomic groups. AGR and BMatMG were log 10 transformed before OLS were performed, presented are the back transformed models (log10 AGR = log10 intercept+log10 BMatMG * slope). Models are ordered by values of intercepts. N = sample size. CI = confidence interval. AIC = Akaike Information Criterion. (DOCX) [file pone.0088834.s003.docx]

**Table S2.** Ordinary linear least square (OLS) regression models of maximum absolute growth rate per day (AGR) on body mass at maximum growth (BMatMG, in gram) for different taxonomic groups. AGR and BMatMG were log 10 transformed before OLS were performed, presented are the back transformed models (log10 A$GR=intercept+log10 BMatMG*slope$). Models are ordered by values of intercepts. N = sample size. CI = confidence interval. AIC = Akaike Information Criterion.

| **group** | **N** | **intercept** | **95% CI** | **p-value** | **slope** | **95% CI** | **p-value** | **R²** | **AIC** |
| --- | --- | --- | --- | --- | --- | --- | --- | --- | --- |
| altrical birds | 380 | -0.436 | [-0.470, -0.401] | <2e-16 | 0.743 | [0.725, 0.762] | <2e-16 | 0.944 | -327.225 |
| precocial birds | 194 | -0.694 | [-0.794, -0.594] | <2e-16 | 0.770 | [0.727, 0.812] | <2e-16 | 0.870 | -96.466 |
| eutherians | 319 | -1.014 | [-1.073, -0.954] | <2e-16 | 0.694 | [0.671, 0.716] | <2e-16 | 0.922 | 87.219 |
| eutherians without Primates + Pinnipedia | 293 | -1.049 | [-1.099, -0.999] | <2e-16 | 0.729 | [0.710, 0.749] | <2e-16 | 0.951 | -38.244 |
| marsupials | 21 | -1.256 | [-1.494, -1.018] | <2e-09 | 0.756 | [0.674, 0.838] | <2e-13 | 0.951 | -14.189 |
| reptiles | 49 | -1.963 | [-2.176, -1.750] | <2e-16 | 0.671 | [0.590, 0.751] | <2e-16 | 0.856 | 61.255 |
| fish | 109 | -2.358 | [-2.461, -2.254] | <2e-16 | 0.782 | [0.741, 0.823] | <2e-16 | 0.931 | 19.107 |
| dinosaurs | 19 | -1.872 | [-2.175, -1.569] | <3e-10 | 0.775 | [0.724, 0.825] | <2e-16 | 0.984 | -15.300 |
