# Supplementary material for: Allometries of Maximum Growth Rate versus Body Mass at Maximum Growth Indicate That Non-Avian Dinosaurs Had Growth Rates Typical of Fast Growing Ectothermic Sauropsids
Source: PLoS One. 2014 Feb 25;9(2):e88834. doi: 10.1371/journal.pone.0088834 (PMC3934860; doi:10.1371/journal.pone.0088834)
Supplement: Table S3 — Ordinary linear least square (OLS) regression models of maximum absolute growth rate per day (AGR) on body mass at maximum growth (BMatMG, in gram) for different taxonomic groups but with a fixed slope of 0.75. AGR and BMatMG were log 10 transformed before regressions were performed (log10 AGR = log10 intercept+log10 BMatMG * 0.75). Regression models are ordered by values of intercepts. N = sample size. CI = confidence interval. AIC = Akaike Information Criterion. (DOCX) [file pone.0088834.s004.docx]

**Tabel S3.** OLS regression models of maximum absolute growth rate per day (AGR) on body mass at maximum growth (BMatMG, in gram) for different taxonomic groups but with a fixed slope of 0.75. AGR and BMatMG were log 10 transformed before regressions were performed ($\log AGR=\log intercept+\log BMatMG*0.75$). Regression models are ordered by values of intercepts. N = sample size. CI = confidence interval. AIC = Akaike Information Criterion.

| **group** | **N** | **intercept** | **95% CI** | **p-value** | **AIC** | **10^intercept** |
| --- | --- | --- | --- | --- | --- | --- |
| altrical birds | 380 | -0.447 | [-0.463, -0.431] | <2e-16 | -328.688 | 0.357 |
| precocial birds | 194 | -0.649 | [-0.675, -0.623] | <2e-16 | -97.615 | 0.224 |
| eutherians without Primates + Pinnipedia | 293 | -1.094 | [-1.120, -1.069] | <2e-16 | -35.810 | 0.081 |
| eutherians | 319 | -1.144 | [-1.175, -1.112] | <2e-16 | 109.193 | 0.072 |
| marsupials | 21 | -1.240 | [-1.306, -1.175] | <2e-16 | -16.165 | 0.058 |
| non-avian dinosaurs | 19 | -1.728 | [-1.793, -1.662] | <2e-16 | -16.138 | 0.019 |
| reptiles | 49 | -2.133 | [-2.259, -2.008] | <2e-16 | 63.186 | 0.007 |
| fish | 109 | -2.286 | [-2.335, -2.237] | <2e-16 | 19.548 | 0.005 |
