# Supplementary material for: Allometries of Maximum Growth Rate versus Body Mass at Maximum Growth Indicate That Non-Avian Dinosaurs Had Growth Rates Typical of Fast Growing Ectothermic Sauropsids
Source: PLoS One. 2014 Feb 25;9(2):e88834. doi: 10.1371/journal.pone.0088834 (PMC3934860; doi:10.1371/journal.pone.0088834)
Supplement: Table S4 — Correlation of regression coefficients (intercept and slope) of the PGLS regression models. For more information on the PGLS regression models see Table 2 in the manuscript. (DOCX) [file pone.0088834.s005.docx]

**Table S4. Correlation of regression coefficients (intercept and slope) of the PGLS regression models.** For more information on the PGLS regression models see Table 2 in the manuscript.

| **group** | **N** | **Correlation of regression coeffiecents (intercept and slope)** |
| --- | --- | --- |
| altrical birds | 343 | -0.669 |
| precocial birds | 164 | -0.568 |
| eutherians | 299 | -0.611 |
| marsupials | 21 | -1.000 |
| dinosaurs | 19 | -0.916 |
| reptiles | 35 | -0.427 |
| fish | 30 | -0.443 |
