# Supplementary material for: Allometries of Maximum Growth Rate versus Body Mass at Maximum Growth Indicate That Non-Avian Dinosaurs Had Growth Rates Typical of Fast Growing Ectothermic Sauropsids
Source: PLoS One. 2014 Feb 25;9(2):e88834. doi: 10.1371/journal.pone.0088834 (PMC3934860; doi:10.1371/journal.pone.0088834)
Supplement: Phylogenetic Information S1 — Phylogenetic information used in PGLS regression analyses. Newick format. (DOCX) [file pone.0088834.s006.docx]

Phylogenetic information S1

**Phylogenetic information used in PGLS regression analyses for altricial birds (Newick format):**

((((((((Falco_sparverius:11.16619733,((Falco_peregrinus:8.487674859,Falco_chicquera:8.487674859):1.383604255,Falco_subbuteo:9.871279113):1.294918215):1.229407707,(Falco_tinnunculus:1.208557017,Falco_cenchroides:1.208557017):11.18704802):1.057466764,Falco_columbarius:13.4530718):62.19089267,((((((Agapornis_personatus:6.821046607,Agapornis_roseicollis:6.821046607):18.33234368,Melopsittacus_undulatus:25.15339028):0.8603496416,Prosopeia_tabuensis:26.01373992):4.339796661,(((Forpus_passerinus:23.38611245,((((Ara_ararauna:2.430634327,Ara_militaris:2.430634327):4.206578064,((Ara_chloropterus:2.836562349,Ara_macao:2.836562349):2.377177036,Ara_rubrogenys:5.213739385):1.423473006):1.888992152,Primolius_auricollis:8.526204543):4.426598381,Enicognathus_ferrugineus:12.95280293):10.43330953):1.128408304,(((Amazona_aestiva:1.718626054,(Amazona_oratrix:0.4263356559,Amazona_ochrocephala:0.4263356559):1.292290398):4.468517656,((Amazona_viridigenalis:2.049480707,Amazona_finschi:2.049480707):2.667449825,Amazona_dufresniana:4.716930532):1.470213177):17.30858763,Myiopsitta_monachus:23.49573134):1.018789422):0.7886822148,Bolborhynchus_lineola:25.30320297):5.050333614):12.31442222,(Calyptorhynchus_funereus:30.52334756,((Cacatua_haematuropygia:8.788834079,Cacatua_sanguinea:8.788834079):2.314700335,(Cacatua_alba:6.479950164,Cacatua_sulphurea:6.479950164):4.62358425):19.41981315):12.14461124):32.11784507,((((Myiobius_atricaudus:25.57944431,((Pyrrhomyias_cinnamomeus:17.39319153,((Elaenia_chiriquensis:7.037404749,Elaenia_flavogaster:7.037404749):9.355450573,(((Myiodynastes_maculatus:7.704781554,((Tyrannus_tyrannus:4.730834182,Tyrannus_melancholicus:4.730834182):0.4605268379,(Tyrannus_verticalis:1.325421755,Tyrannus_forficatus:1.325421755):3.865939265):2.513420534):1.164374593,(Myiozetetes_cayanensis:4.208147456,Myiozetetes_similis:4.208147456):4.661008691):5.506553899,(Empidonax_traillii:5.864915296,Empidonax_difficilis:5.864915296):8.51079475):2.017145276):1.00033621):5.668466229,((Todirostrum_cinereum:18.01063562,(Rhynchocyclus_olivaceus:12.75694508,Tolmomyias_sulphurescens:12.75694508):5.253690539):0.8860806628,Mionectes_macconnelli:18.89671629):4.164941475):2.517786548):0.7689048963,(Procnias_averano:23.51489362,Pipra_mentalis:23.51489362):2.833455583):20.66651042,(((Sakesphorus_canadensis:9.616348342,Thamnophilus_doliatus:9.616348343):2.751154836,(Cercomacra_tyrannina:11.97493004,Myrmeciza_exsul:11.97493004):0.3925731407):0.4391181943,Myrmotherula_axillaris:12.80662137):34.20823826):17.54669295,(Sericulus_chrysocephalus:55.03412603,((Ramsayornis_modestus:39.4976721,Gerygone_igata:39.4976721):13.54998098,(Pomatostomus_temporalis:50.57611616,((Vireo_flavoviridis:37.78639634,((Melampitta_lugubris:26.09511561,Chasiempis_sandwichensis:26.09511561):4.139859099,((Lanius_ludovicianus:10.70229021,Lanius_collurio:10.70229021):17.59277484,((((Gymnorhinus_cyanocephalus:12.20836919,Aphelocoma_coerulescens:12.20836919):3.695048247,Cyanocorax_beecheii:15.90341744):3.697272129,((Nucifraga_caryocatactes:14.00733399,(Corvus_monedula:10.58558842,(((Corvus_brachyrhynchos:0.6967644167,Corvus_caurinus:0.6967644167):3.473303729,Corvus_corone:4.170068145):3.049528172,((Corvus_cryptoleucus:2.732196702,Corvus_albus:2.732196702):1.286571621,Corvus_capensis:4.018768323):3.200827993):3.365992099):3.421745575):2.398726801,Pica_pica:16.40606079):3.194628775):3.605342527,(Pyrrhocorax_pyrrhocorax:10.73097151,Pyrrhocorax_graculus:10.73097151):12.47506058):5.089032957):1.939909659):7.551421632):12.32650171,(((((Parus_ater:14.40232576,(Parus_atricapillus:10.50708411,Parus_montanus:10.50708411):3.895241656):1.911900861,(Parus_palustris:8.029894798,Parus_major:8.029894798):8.28433183):2.65159495,Parus_caeruleus:18.96582158):23.26272145,(((Eremophila_alpestris:13.93681005,Alauda_arvensis:13.93681005):7.821960038,Certhilauda_albescens:21.75877008):13.12066393,(((Phylloscopus_sibilatrix:14.80813845,(Phylloscopus_collybita:5.557427304,Phylloscopus_trochilus:5.557427304):9.250711147):15.60343805,(((Tachycineta_albilinea:8.955958703,(Tachycineta_thalassina:7.733081942,Tachycineta_bicolor:7.733081942):1.222876761):5.392959853,((Stelgidopteryx_ruficollis:11.65061696,(Progne_chalybea:4.11611995,Progne_subis:4.116119951):7.534497014):1.60619667,Pygochelidon_cyanoleuca:13.25681363):1.092104922):4.498137374,(((Hirundo_rustica:6.410903166,Hirundo_tahitica:6.410903166):9.212976471,Petrochelidon_pyrrhonota:15.62387964):0.424827063,Delichon_urbicum:16.0487067):2.798349229):11.56452057):2.803354715,((Acrocephalus_scirpaceus:9.991363791,Acrocephalus_arundinaceus:9.991363791):17.20038921,(Sylvia_borin:13.2123217,Sylvia_atricapilla:13.2123217):13.97943131):6.023178203):1.664502802):7.349109012):3.364084912,((((((Campylorhynchus_brunneicapillus:16.03495195,(Cistothorus_palustris:10.79560585,(Troglodytes_troglodytes:10.61438465,Troglodytes_aedon:10.61438465):0.1812212026):5.239346095):8.400409624,Microbates_collaris:24.43536157):14.39505074,(((Sturnus_vulgaris:21.85310585,((Mimus_polyglottos:12.30523268,(Toxostoma_longirostre:8.036705889,Toxostoma_curvirostre:8.036705888):4.268526797):2.666152573,Margarops_fuscatus:14.97138526):6.881720598):1.139922746,Buphagus_erythrorhynchus:22.9930286):5.078526543,(((Sialia_sialis:18.10179703,Myadestes_ralloides:18.10179703):1.54613987,((((Turdus_leucomelas:5.179909718,Turdus_grayi:5.179909717):3.350040237,(Turdus_migratorius:8.11009954,Turdus_pilaris:8.11009954):0.4198504152):0.44548766,Turdus_merula:8.975437615):7.378690518,((((Catharus_minimus:1.028813636,Catharus_fuscescens:1.028813636):3.921774051,Catharus_guttatus:4.950587687):2.173116053,Catharus_ustulatus:7.12370374):5.248392467,Hylocichla_mustelina:12.37209621):3.982031927):3.293808766):3.898258378,((Erithacus_rubecula:15.17619791,(Ficedula_hypoleuca:14.250003,(Phoenicurus_phoenicurus:13.28871642,((Saxicola_torquatus:8.62517848,Saxicola_rubetra:8.625178479):3.270086864,(Myrmecocichla_formicivora:9.794362316,Oenanthe_oenanthe:9.794362315):2.100903027):1.393451075):0.9612865841):0.9261949057):1.326779932,Pogonocichla_stellata:16.50297784):7.043217439):4.525359867):10.75885716):1.809272686,Bombycilla_cedrorum:40.63968499):3.94623413,(Regulus_ignicapilla:29.56831621,Regulus_regulus:29.56831621):15.01760291):0.5098343025,(((((((Euphonia_violacea:11.04471232,(Euphonia_laniirostris:4.988702009,Euphonia_minuta:4.988702009):6.056010312):11.49308742,((Leucosticte_tephrocotis:15.43415915,Loxioides_bailleui:15.43415915):1.541501511,((Carduelis_spinus:8.625886184,Serinus_serinus:8.625886185):0.07880839989,Carduelis_flammea:8.704694585):8.270966079):5.562139073):3.47165509,(((Cardinalis_cardinalis:19.70858393,(((Thraupis_palmarum:3.826954993,Thraupis_episcopus:3.826954993):6.191059587,Tangara_inornata:10.01801458):3.551019667,(Saltator_albicollis:12.06479856,(((Sporophila_nigricollis:4.291777621,(Sporophila_castaneiventris:3.860048074,Sporophila_corvina:3.860048074):0.4317295469):7.086423319,Cyanerpes_cyaneus:11.37820094):0.3038447848,(Ramphocelus_carbo:10.58851673,Volatinia_jacarina:10.58851673):1.093528992):0.3827528318):1.50423569):6.139549682):1.805503555,((((((Spizella_pusilla:2.90778404,Spizella_breweri:2.907784041):1.076449853,Spizella_passerina:3.984233893):5.985058473,(((Pipilo_erythrophthalmus:7.193691518,Pipilo_aberti:7.193691517):0.8444764841,((Melospiza_melodia:5.255942197,Passerculus_sandwichensis:5.255942197):1.588052248,((Ammodramus_maritimus:2.063849029,Ammodramus_caudacutus:2.063849029):3.750532503,(Amphispiza_belli:4.957116286,Pooecetes_gramineus:4.957116286):0.8572652457):1.029612914):1.194173556):0.425295371,(Zonotrichia_capensis:3.849825375,((Zonotrichia_leucophrys:0.3467619845,Zonotrichia_atricapilla:0.3467619845):1.443418448,Zonotrichia_albicollis:1.790180433):2.059644942):4.613637998):1.505828993):0.6380316086,(Aimophila_carpalis:7.885215192,(Ammodramus_aurifrons:6.774525298,Arremonops_conirostris:6.774525298):1.110689894):2.722108783):1.414087882,((((Molothrus_ater:5.935598343,(Agelaius_xanthomus:4.794594425,Agelaius_phoeniceus:4.794594425):1.141003917):0.2532321752,(Euphagus_cyanocephalus:4.37329628,(Quiscalus_niger:1.001126013,(Quiscalus_mexicanus:0.821728568,Quiscalus_major:0.821728568):0.1793974454):3.372170266):1.815534237):4.708172835,Psarocolius_wagleri:10.89700335):1.124288291,Xanthocephalus_xanthocephalus:12.02129164):0.0001202141186):6.962831306,(Seiurus_aurocapilla:11.32038877,((Geothlypis_trichas:8.923490091,Seiurus_motacilla:8.923490092):0.3266834202,(((Dendroica_kirtlandii:4.619460533,Dendroica_petechia:4.619460534):0.6308815056,Setophaga_ruticilla:5.250342039):3.684740316,Myioborus_miniatus:8.935082355):0.3150911565):2.070215261):7.66385439):2.529844322):1.397907701,((Calcarius_lapponicus:15.14130629,Calcarius_mccownii:15.14130628):1.807287187,Plectrophenax_nivalis:16.94859347):5.963401713):3.097459639):1.572270371,(Anthus_spinoletta:19.69602951,Motacilla_cinerea:19.69602951):7.885695685):1.676632182,Passer_domesticus:29.25835738):4.201506146,(Amandava_amandava:14.55627432,(Taeniopygia_guttata:11.91122153,Lonchura_striata:11.91122153):2.645052788):18.9035892):4.655267209,((Nectarinia_jugularis:13.5390498,Nectarinia_talatala:13.5390498):4.282572887,Nectarinia_osea:17.82162268):20.29350805):6.980622695):0.4968745116):4.52027011):0.4632181107):2.471536924):1.986472953):9.527426537):10.2242513):0.8581606009):0.7957190894,Urocolius_macrourus:76.43968356):1.969856678,((((Merops_viridis:62.73392531,Coracias_caudatus:62.7339253):6.889839983,(Ramphastos_dicolorus:33.14248555,(Dryocopus_pileatus:15.42164894,(Picoides_borealis:9.872240841,(Dendrocopos_leucotos:2.643123189,Dendrocopos_major:2.643123189):7.229117651):5.5494081):17.72083661):36.48127974):6.317893203,(Tyto_alba:69.54289238,(Otus_senegalensis:33.4849256,((Megascops_asio:26.27656413,(Strix_uralensis:23.66857032,((Bubo_virginianus:13.05898322,Bubo_bubo:13.05898322):4.830382837,Bubo_lacteus:17.88936606):5.779204266):2.607993809):2.931940946,Asio_otus:29.20850508):4.276420523):36.05796677):6.398766117):1.739492436,(((Sarcoramphus_papa:38.84087401,Vultur_gryphus:38.84087401):16.05348026,(Coragyps_atratus:40.89196108,Cathartes_aura:40.89196108):14.00239319):22.39895448,(((Neophron_percnopterus:34.41606294,(((Trigonoceps_occipitalis:12.6797418,(Necrosyrtes_monachus:10.63172765,(Gyps_africanus:3.0693573,Gyps_coprotheres:3.0693573):7.562370351):2.048014146):12.34327587,(Terathopius_ecaudatus:17.60804215,Circaetus_cinereus:17.60804215):7.414975519):3.265461433,((((Accipiter_nisus:16.80890316,Accipiter_fasciatus:16.80890316):3.322888199,(((Accipiter_gentilis:3.075324769,Accipiter_minullus:3.07532477):9.242887065,Accipiter_cooperii:12.31821184):5.625030656,(Circus_cyaneus:9.313799258,Circus_ranivorus:9.313799257):8.629443233):2.188548871):4.054998607,((Ictinia_mississippiensis:13.13617722,((Buteo_jamaicensis:3.493938751,(Buteo_swainsoni:3.221984721,((Buteo_oreophilus:0.2586808878,Buteo_buteo:0.2586808878):1.794732955,Buteo_regalis:2.053413843):1.168570878):0.2719540304):1.047301607,Buteo_platypterus:4.541240358):8.594936859):6.055039285,(Milvus_migrans:16.39218822,(Haliaeetus_vocifer:11.48738351,(Haliaeetus_leucocephalus:1.807746051,Haliaeetus_albicilla:1.807746051):9.679637461):4.904804705):2.799028285):4.995573465):2.675471587,(Stephanoaetus_coronatus:16.36927801,(Lophaetus_occipitalis:8.995796695,(Aquila_wahlbergi:7.970613503,((Aquila_verreauxii:6.451357034,Aquila_chrysaetos:6.451357034):0.880296126,Aquila_rapax:7.33165316):0.6389603431):1.025183192):7.373481321):10.49298354):1.426217544):6.127583841):15.19614407,Pandion_haliaetus:49.61220701):0.0004961270314,Sagittarius_serpentarius:49.61270314):27.68060562):0.3878421733):0.7283893128):1.165013295,((Phaethon_aethereus:25.52248161,(Phaethon_rubricauda:11.38057694,Phaethon_lepturus:11.38057694):14.14190467):49.2774163,((((Columbina_talpacoti:3.576096464,Columbina_passerina:3.576096464):29.15069032,((Geopelia_cuneata:28.69851769,(Ptilinopus_superbus:27.30961378,Oena_capensis:27.30961377):1.388903917):1.387936111,((((Columba_oenas:6.533761924,Columba_livia:6.533761924):1.566136999,Columba_guinea:8.099898923):2.153201269,Columba_palumbus:10.25310019):5.797660978,Streptopelia_decaocto:16.05076117):14.03569263):2.640332979):35.6118646,Opisthocomus_hoazin:68.33865138):6.151424868,(((((Pygoscelis_papua:7.328956473,Pygoscelis_antarcticus:7.328956473):9.512664402,Pygoscelis_adeliae:16.84162087):8.526592113,((Aptenodytes_patagonicus:6.521263192,Aptenodytes_forsteri:6.521263192):16.29225883,((Spheniscus_demersus:1.873972273,Spheniscus_magellanicus:1.873972273):14.47187112,(Megadyptes_antipodes:8.526309452,(Eudyptes_pachyrhynchus:4.339863021,Eudyptes_chrysocome:4.33986302):4.186446433):7.819533938):6.467678631):2.554690966):38.46650504,((((Ciconia_abdimii:21.8334173,(Ciconia_ciconia:13.04843981,Ciconia_maguari:13.04843981):8.784977493):1.643060969,Leptoptilos_crumeniferus:23.47647827):5.958204161,Mycteria_americana:29.43468243):33.25847701,(((((Eudocimus_albus:38.8018381,Geronticus_eremita:38.80183809):8.720630982,Platalea_leucorodia:47.52246908):4.124963447,(((Egretta_caerulea:12.84525118,Egretta_garzetta:12.84525118):18.01432229,(Butorides_virescens:29.18352154,((Ardea_purpurea:7.712765773,(Ardea_cinerea:2.440034693,Ardea_herodias:2.440034693):5.27273108):9.342977737,Bubulcus_ibis:17.05574351):12.12777803):1.67605193):6.087687816,Botaurus_stellaris:36.94726128):14.70017124):7.507397807,((Pelecanus_rufescens:37.41894198,Pelecanus_occidentalis:37.41894198):12.95194454,Scopus_umbretta:50.37088652):8.783943814):0.6295444366,((((Fregata_aquila:6.815012329,Fregata_magnificens:6.815012329):6.038274102,Fregata_ariel:12.85328643):9.601041769,Fregata_minor:22.4543282):31.23147274,(((Morus_serrator:6.542279707,Morus_bassanus:6.542279707):11.82180152,((Sula_dactylatra:7.500095036,Sula_leucogaster:7.500095036):3.844650597,Sula_sula:11.34474563):7.01933559):19.31267214,(Phalacrocorax_aristotelis:11.44481027,(Phalacrocorax_carbo:8.873564864,Phalacrocorax_auritus:8.873564864):2.571245404):26.2319431):16.00904757):6.098573829):2.908784668):1.141558594):7.61605948,(((Carpococcyx_renauldi:58.53259681,Centropus_senegalensis:58.5325968):3.841883515,(Clamator_glandarius:47.56791061,((Chrysococcyx_lucidus:0.9447366038,Chrysococcyx_basalis:0.9447366038):24.88032148,Chrysococcyx_caprius:25.82505808):21.74285252):14.80656971):5.18738794,Crotophaga_ani:67.56186825):3.888909254):3.039298739):0.3098216579):4.774655627):0.03928657439,(Steatornis_caripensis:74.26705394,((((((Calliphlox_evelynae:6.33770647,Selasphorus_rufus:6.33770647):10.03800577,(Chlorostilbon_mellisugus:14.89009807,((Amazilia_fimbriata:7.495803988,Amazilia_tobaci:7.495803987):1.122191068,Amazilia_tzacatl:8.617995055):6.272103012):1.485614173):2.867366624,Oxypogon_guerinii:19.24307887):3.623455145,Colibri_coruscans:22.86653401):3.119638719,Phaethornis_ruber:25.98617273):32.12595694,((Streptoprocne_rutila:27.55877518,(Cypseloides_cherriei:20.40772151,Cypseloides_cryptus:20.40772151):7.151053681):19.02228887,(((Chaetura_vauxi:6.030842618,Chaetura_pelagica:6.030842618):7.506450918,Chaetura_brachyura:13.53729354):21.95721355,((Collocalia_esculenta:26.25450309,(Collocalia_leucophaea:14.68080803,Collocalia_fuciphaga:14.68080803):11.57369506):4.909476639,(Cypsiurus_parvus:22.16723092,(Tachymarptis_melba:16.64817943,(Apus_apus:13.01668062,Apus_pacificus:13.01668062):3.631498806):5.519051495):8.996748809):4.330527351):11.08655698):11.53106561):16.15492427):5.346786169);

References

Jetz W, Thomas GH, Joy JB, Hartmann K, Mooers AO (2012) The global diversity of birds in space and time. Nature 491: 444-448.

**Phylogenetic information used in PGLS regression analyses for precocial birds (Newick format):**

(((Eudromia_elegans:64.65146208,((Nothura_darwinii:14.67619791,Nothura_maculosa:14.67619791):32.44879506,Rhynchotus_rufescens:47.12499298):17.52646911):21.23985938,Apteryx_australis:85.89132145):27.35838974,(((((Callipepla_californica:14.72310057,Cyrtonyx_montezumae:14.72310057):11.70738509,((((Meleagris_gallopavo:13.33200564,(((Lagopus_muta:2.781935985,Lagopus_lagopus:2.781935985):3.636873292,((Dendragapus_obscurus:3.238948074,Tympanuchus_phasianellus:3.238948074):2.68985728,(Dendragapus_canadensis:5.029422762,(Tetrao_urogallus:3.641452726,Tetrao_tetrix:3.641452726):1.387970035):0.8993825931):0.4900039212):3.698305541,(Bonasa_umbellus:9.488840514,Bonasa_bonasia:9.488840514):0.6282743028):3.214890821):1.28021525,(Phasianus_colchicus:13.03514847,Perdix_perdix:13.03514847):1.577072418):3.528722966,(Gallus_gallus:16.98479766,Pavo_cristatus:16.98479766):1.156146199):0.6103865046,((Coturnix_delegorguei:8.433634685,(Coturnix_japonica:0.5162438304,Coturnix_coturnix:0.5162438304):7.917390855):2.730041489,Coturnix_chinensis:11.16367617):7.587654184):7.679155305):30.15051882,(Macrocephalon_maleo:34.50619364,(Alectura_lathami:27.15659061,Leipoa_ocellata:27.15659061):7.349603028):22.07481084):15.78303557,(Dendrocygna_autumnalis:33.12811224,((Cygnus_olor:13.53550123,(Anser_anser:1.35946045,Anser_indicus:1.35946045):12.17604078):10.56201587,(((Anas_platyrhynchos:0.5183891299,Anas_rubripes:0.5183891299):5.248118221,Anas_strepera:5.766507351):6.547638511,(((Aythya_valisineria:1.639816738,Aythya_americana:1.639816738):0.4699963829,(Aythya_affinis:1.369422518,Aythya_fuligula:1.369422518):0.7403906031):8.828960093,(((Chloephaga_picta:8.731822028,(Tadorna_tadorna:2.811077243,Tadorna_ferruginea:2.811077243):5.920744785):0.8445375387,Aix_galericulata:9.576359567):1.06521775,(Somateria_mollissima:9.542623667,Mergus_serrator:9.542623666):1.09895365):0.2971958982):1.375372647):11.78337124):9.030595129):39.23592781):25.19656036,(((((Turnix_suscitator:60.00831048,((((Anous_stolidus:8.384380122,Anous_minutus:8.384380122):13.34722638,(((Sterna_albifrons:13.7139333,(((((Sterna_paradisaea:1.491344731,Sterna_vittata:1.491344731):2.276614786,Sterna_hirundo:3.767959517):0.5897342765,(Sterna_dougallii:2.924617481,Sterna_sumatrana:2.924617481):1.433076313):2.043532577,Sterna_forsteri:6.401226371):2.602597738,Chlidonias_niger:9.003824108):4.71010919):1.423861719,((Sterna_lunata:3.022876591,Sterna_anaethetus:3.022876591):3.286985414,Sterna_fuscata:6.309862005):8.827933011):4.394584289,Gygis_alba:19.5323793):2.199227195):0.7186319611,((Creagrus_furcatus:9.81584216,(((((Larus_occidentalis:1.212713881,((Larus_argentatus:0.7619557493,Larus_marinus:0.7619557493):0.1900709123,((Larus_glaucescens:0.595648656,Larus_californicus:0.5956486561):0.09096488755,Larus_fuscus:0.6866135436):0.265413118):0.2606872199):0.8855931464,Larus_canus:2.098307028):3.428102087,Larus_ridibundus:5.526409115):1.808859544,Larus_minutus:7.335268659):0.3794473344,Rissa_tridactyla:7.714715995):2.101126166):10.5026087,Rynchops_niger:20.31845086):2.131787599):2.979432735,((Stercorarius_longicaudus:9.410137231,(Catharacta_skua:2.922859104,Catharacta_maccormicki:2.922859104):6.487278128):13.80483201,(((Fratercula_arctica:9.267853585,Cerorhinca_monocerata:9.267853585):8.164910815,(((Aethia_psittacula:4.331898069,Aethia_pusilla:4.331898069):1.325187769,Aethia_cristatella:5.657085837):1.852522443,Ptychoramphus_aleuticus:7.50960828):9.92315612):1.832975591,(((Uria_lomvia:5.044281523,Uria_aalge:5.044281523):7.253858348,(Alca_torda:11.22082381,Alle_alle:11.22082381):1.077316065):4.61236804,(Cepphus_columba:6.775171727,Cepphus_grylle:6.775171727):10.13533618):2.35523208):3.94922925):2.214701956):34.57863927):3.374560563,(Pedionomus_torquatus:58.76282085,(Numenius_arquata:44.00565491,(((Arenaria_interpres:30.0591723,(Philomachus_pugnax:20.13779687,((Calidris_alpina:14.50230606,((Calidris_alba:10.23754188,Calidris_fuscicollis:10.23754188):3.025110464,Calidris_pusilla:13.26265234):1.23965372):1.637894296,(Calidris_bairdii:14.37310769,Calidris_melanotos:14.37310769):1.767092667):3.997596511):9.921375429):6.638261566,((Gallinago_gallinago:18.1543835,Coenocorypha_pusilla:18.15438351):17.57809208,Tringa_totanus:35.73247559):0.9649582778):2.978463784,Limosa_limosa:39.67589764):4.32975726):14.75716594):4.620050187):4.469464416,((Pluvianus_aegyptius:56.2262983,(Pluvialis_apricaria:48.06921105,(((Eudromias_morinellus:30.73025965,((Charadrius_melodus:9.985565933,Charadrius_hiaticula:9.985565933):16.89480861,Charadrius_dubius:26.88037454):3.849885101):7.069112542,Vanellus_vanellus:37.79937219):7.183789668,((Haematopus_ostralegus:17.43514592,Haematopus_moquini:17.43514592):11.81946529,(Recurvirostra_avosetta:20.33799585,(Himantopus_himantopus:4.213145575,Himantopus_novaezelandiae:4.213145575):16.12485028):8.916615351):15.72855065):3.086049201):8.157087248):4.311320586,Burhinus_oedicnemus:60.5376189):7.31471656):11.60988198,(Picathartes_oreas:7.596428996,Picathartes_gymnocephalus:7.596428996):71.86578843):0.1123361038,(((Oceanites_oceanicus:53.84654948,((((Pagodroma_nivea:12.50608898,Thalassoica_antarctica:12.50608898):4.003999768,((Fulmarus_glacialoides:9.928724189,(Macronectes_halli:1.946273085,Macronectes_giganteus:1.946273085):7.982451104):2.487098682,Daption_capense:12.41582287):4.094265876):21.88806881,(((Pelecanoides_georgicus:15.59764051,Pelecanoides_urinatrix:15.59764051):19.28734889,(Halobaena_caerulea:17.92821364,(Pachyptila_turtur:5.721010797,(Pachyptila_salvini:0.9122964159,Pachyptila_desolata:0.9122964159):4.808714381):12.20720285):16.95677575):2.237921714,(((Pterodroma_hypoleuca:15.93881703,Pterodroma_phaeopygia:15.93881703):2.138692905,Pterodroma_macroptera:18.07750993):17.67246638,((Procellaria_cinerea:5.336908307,Procellaria_aequinoctialis:5.336908308):24.26888281,((Puffinus_griseus:9.062466797,Puffinus_pacificus:9.062466796):10.5436616,Calonectris_diomedea:19.60612839):9.99966272):6.144185195):1.3729348):1.275246449):13.86137607,((Phoebetria_palpebrata:15.79957357,(Diomedea_exulans:7.903981478,Thalassarche_chrysostoma:7.903981478):7.89559209):7.971408014,((Phoebastria_nigripes:2.580555761,Phoebastria_immutabilis:2.580555761):11.32676817,(Diomedea_amsterdamensis:5.844195544,Diomedea_epomophora:5.844195544):8.063128388):9.86365765):28.48855204):1.587015856):4.661694518,((Oceanodroma_furcata:30.0979919,(Oceanodroma_leucorhoa:20.36616792,Oceanodroma_homochroa:20.36616792):9.731823977):4.144222366,Oceanodroma_castro:34.24221427):24.26602973):12.94253351,(Otis_tarda:69.9463993,((((Porzana_carolina:19.30440857,Porzana_porzana:19.30440856):4.013453796,(Fulica_americana:4.484095387,Fulica_atra:4.484095386):18.83376697):9.429704737,(Crex_crex:19.25381423,(Rallus_aquaticus:14.57643392,Rallus_elegans:14.57643392):4.677380303):13.49375287):19.91574035,((((Grus_antigone:2.106637651,Grus_rubicunda:2.106637651):1.011612588,Grus_vipio:3.118250239):4.524622699,(Grus_japonensis:5.058178409,(Grus_americana:3.011397787,(Grus_monacha:2.2480917,Grus_grus:2.2480917):0.7633060868):2.046780622):2.58469453):0.711487093,Grus_leucogeranus:8.354360032):44.30894742):17.28309186):1.504378202):8.123776024):17.98604686):15.68911079);

References

Jetz W, Thomas GH, Joy JB, Hartmann K, Mooers AO (2012) The global diversity of birds in space and time. Nature 491: 444-448.

**Phylogenetic information used in PGLS regression analyses for Eutherians (Newick format):**

((((((((((Napaeozapus_insignis:11.9,Zapus_hudsonius:11.9):58.4,(((Acomys_cahirinus:12.5,Lophuromys_sikapusi:12.5):16.6,(Aethomys_chrysophilus:19.9,Aethomys_hindei:19.9,Aethomys_kaiseri:19.9):9.2,Apodemus_argenteus:29.1,Chiropodomys_gliroides:29.1,(Mesembriomys_gouldii:28.9,(Notomys_alexis:10.9,Notomys_cervinus:10.9,Notomys_mitchellii:10.9):18,(Pseudomys_australis:21.5,Pseudomys_gracilicaudatus:21.5,Pseudomys_higginsi:21.5,Pseudomys_novaehollandiae:21.5):7.4):0.2,Millardia_meltada:29.1,Hydromys_chrysogaster:29.1,Grammomys_dolichurus:29.1,Lemniscomys_striatus:29.1,Leopoldamys_sabanus:29.1,Mastomys_natalensis:29.1,(Mus_musculus:13.8,(Mus_minutoides:7.6,Mus_musculoides:7.6):6.2):15.3,Otomys_irroratus:29.1,((Rattus_fuscipes:9.6,Rattus_lutreolus:9.6):0.2,Rattus_exulans:9.8,Rattus_rattus:9.8):19.3,Rhabdomys_pumilio:29.1,Sundamys_muelleri:29.1,Zelotomys_woosnami:29.1):19,((((Akodon_molinae:16.1,(Calomys_callosus:8.3,Calomys_lepidus:8.3):7.8,Oligoryzomys_eliurus:16.1,Oryzomys_palustris:16.1):6.7,(Sigmodon_hispidus:5.7,Sigmodon_ochrognathus:5.7):17.1):12.1,(Ototylomys_phyllotis:13.5,Tylomys_nudicaudus:13.5):21.4):3.3,(((Baiomys_taylori:21,Ochrotomys_nuttalli:21):3.5,(((Podomys_floridanus:18.7,(Peromyscus_californicus:11.8,Peromyscus_eremicus:11.8,Peromyscus_gossypinus:11.8,Peromyscus_leucopus:11.8,Peromyscus_maniculatus:11.8,Peromyscus_megalops:11.8,Peromyscus_melanocarpus:11.8,Peromyscus_mexicanus:11.8,Peromyscus_oreas:11.8,Peromyscus_polionotus:11.8,Peromyscus_truei:11.8,Peromyscus_yucatanicus:11.8):6.9):1.5,(Onychomys_leucogaster:9.8,Onychomys_torridus:9.8):10.4):1.8,Reithrodontomys_humulis:22):2.5):1.7,((Neotoma_floridana:7.4,Neotoma_micropus:7.4):8.1,Neotoma_cinerea:15.5):10.7):12):9.9,Mesocricetus_auratus:48.1,((Clethrionomys_gapperi:4.7,Clethrionomys_glareolus:4.7,Clethrionomys_rutilus:4.7):14.6,(Arborimus_longicaudus:4.5,Phenacomys_ungava:4.5):14.8,Arvicola_terrestris:19.3,(Dicrostonyx_groenlandicus:5,Dicrostonyx_unalascensis:5):14.3,(Lemmus_lemmus:9.3,Lemmus_sibiricus:9.3):10,(Microtus_arvalis:9,(Microtus_californicus:6.8,Microtus_oregoni:6.8,Microtus_pennsylvanicus:6.8):2.2,Microtus_oeconomus:9,Microtus_ochrogaster:9,Microtus_pinetorum:9,(Microtus_abbreviatus:8,Microtus_miurus:8):1):10.3,Neofiber_alleni:19.3,Ondatra_zibethicus:19.3):28.8,(((Meriones_hurrianae:14.5,(Meriones_crassus:11.4,Meriones_unguiculatus:11.4):3.1):7.1,Psammomys_obesus:21.6):2,Desmodillus_auricularis:23.6,Gerbillus_pyramidum:23.6,(Tatera_indica:21.6,Tatera_brantsii:21.6):2):24.5,Cricetomys_gambianus:48.1,(Cannomys_badius:14.2,Tachyoryctes_ruandae:14.2):33.9):22.2):7.9,(Castor_fiber:69,(((Perognathus_longimembris:31.9,Chaetodipus_californicus:31.9):2.6,((Dipodomys_heermanni:1.7,Dipodomys_stephensi:1.7):4.9,(Dipodomys_merriami:2,Dipodomys_nitratoides:2):4.6):27.9):1.4,(Thomomys_talpoides:24.8,(Heteromys_desmarestianus:10.2,(Liomys_salvini:5.6,Liomys_pictus:5.6):4.6):14.6):11.1):33.1):9.2):4.4,((Octodon_degus:46.7,(Massoutiera_mzabi:9.2,Pectinator_spekei:9.2,Ctenodactylus_vali:9.2):37.5):0.2,(Hydrochaeris_hydrochaeris:43.8,Abrocoma_cinerea:43.8,(Cavia_porcellus:14.7,Cavia_aperea:14.7,Microcavia_australis:14.7,Galea_musteloides:14.7):29.1,((Hoplomys_gymnurus:17.4,Proechimys_guairae:17.4,Proechimys_semispinosus:17.4):3.6,((Capromys_pilorides:6.8,Geocapromys_ingrahami:6.8):12.5,Plagiodontia_aedium:19.3):1.7):22.8,Agouti_paca:43.8,Atherurus_africanus:43.8,Heterocephalus_glaber:43.8,(Erethizon_dorsatum:10.9,Coendou_prehensilis:10.9):32.9):3.1):35.7):0.2,((Aplodontia_rufa:56.4,(((((((((((Spermophilus_undulatus:1.4,Spermophilus_columbianus:1.4):1.1,(Spermophilus_richardsonii:2.2,Spermophilus_armatus:2.2):0.3):1,(Spermophilus_tridecemlineatus:2.8,Spermophilus_franklinii:2.8):0.7):0.4,Spermophilus_beldingi:3.9):0.5,Spermophilus_tereticaudus:4.4):3,Spermophilus_lateralis:7.4):0.7,(Cynomys_ludovicianus:0.7,Cynomys_mexicanus:0.7):7.4):3.8,Ammospermophilus_leucurus:11.9):14.3,(((Tamias_amoenus:9.1,(Tamias_quadrivittatus:2.7,Tamias_palmeri:2.7):6.4):0.6,Tamias_townsendii:9.7):0.5,Tamias_panamintinus:10.2):16):5,Paraxerus_cepapi:31.2):10.3,(((Tamiasciurus_hudsonicus:14.2,((Sciurus_carolinensis:2,Sciurus_niger:2):2.3,Sciurus_vulgaris:4.3):9.9):8.9,Glaucomys_sabrinus:23.1):7,Funambulus_pennantii:30.1):11.4):14.9):17.6,Eliomys_quercinus:74):8.8):6.1,(((Sylvilagus_aquaticus:8,Sylvilagus_floridanus:8):16.8,(((Lepus_californicus:6,Lepus_americanus:6):1.8,(Lepus_othus:7.7,Lepus_europaeus:7.7):0.1):16.2,Oryctolagus_cuniculus:24):0.8):39.5,(Ochotona_princeps:13.3,Ochotona_rufescens:13.3):51):24.6):2.9,((((((Macaca_mulatta:8.5,Macaca_nemestrina:8.5):11.2,Nasalis_larvatus:19.7):14.7,(((Gorilla_gorilla:11.5,(Homo_sapiens:8.6,Pan_troglodytes:8.6):2.9):6.5,Pongo_pygmaeus:18):3.4,Hylobates_lar:21.4):13):16.6,((((Callimico_goeldii:12.7,(Callithrix_argentata:5.6,Callithrix_jacchus:5.6):7.1):1.8,Leontopithecus_rosalia:14.5):1.2,(Saguinus_oedipus:8.6,Saguinus_nigricollis:8.6):7.1):4.5,Saimiri_sciureus:20.2):30.8):33.5,(Microcebus_murinus:72.8,((Arctocebus_calabarensis:18.7,Perodicticus_potto:18.7):20.2,Galago_senegalensis:38.9):33.9):11.7):7.1,Tupaia_belangeri:91.6):0.2):4.3,(((((((((((((Connochaetes_taurinus:13.8,Damaliscus_pygargus:13.8):7.8,((Ammotragus_lervia:15.4,(Ovis_canadensis:14.5,(Capra_hircus:7.3,Capra_ibex:7.3):7.2):0.9):0.7,Ovibos_moschatus:16.1):5.5):1.4,Aepyceros_melampus:23,(Antidorcas_marsupialis:10.7,Saiga_tatarica:10.7):12.3,Redunca_fulvorufula:23,Madoqua_kirkii:23):4.5,(Syncerus_caffer:22.7,(Taurotragus_oryx:18.2,Tragelaphus_scriptus:18.2,Tragelaphus_imberbis:18.2):4.5):4.8):0.2,(Alces_alces:16.5,((Odocoileus_virginianus:4.2,Pudu_puda:4.2):7.3,Rangifer_tarandus:11.5):5):11.2):0.1,Antilocapra_americana:27.8):33.8,Phocoena_phocoena:61.6):6.2,(Potamochoerus_porcus:21.9,Sus_scrofa:21.9):45.9):2.9,Vicugna_vicugna:70.7):12.7,(Diceros_bicornis:55.8,Equus_burchellii:55.8):27.6):1.2,((((((((((Mustela_putorius:9.9,Mustela_nivalis:9.9):12.9,(Gulo_gulo:16.9,Eira_barbara:16.9):5.9,Ictonyx_striatus:22.8,Poecilogale_albinucha:22.8):1.3,Meles_meles:24.1,(Mephitis_mephitis:9.5,Spilogale_putorius:9.5):14.6):5.5,((Procyon_lotor:15.1,Bassariscus_astutus:15.1):10.2,Potos_flavus:25.3):4.3):7.9,Ailurus_fulgens:37.5):9.3,((Odobenus_rosmarus:24.1,(Arctocephalus_gazella:9.5,Callorhinus_ursinus:9.5):14.6):8.6,(Phoca_vitulina:22.1,Mirounga_leonina:22.1):10.6):14.1):2.9,((Ursus_maritimus:11.4,Ursus_americanus:11.4):24.2,Ailuropoda_melanoleuca:35.6):14.1):6.8,((((Canis_lupus:1.4,Canis_latrans:1.4):0.2,Canis_mesomelas:1.6):1.9,Cerdocyon_thous:3.5,Chrysocyon_brachyurus:3.5):3.6,((Vulpes_vulpes:1.8,Vulpes_velox:1.8):5,Urocyon_cinereoargenteus:6.8):0.3):49.4):6.9,((((((((Panthera_leo:5.7,Panthera_pardus:5.7):0.2,Panthera_onca:5.9):4.8,(Lynx_lynx:8.7,Lynx_rufus:8.7):2):0.2,Profelis_aurata:10.9):0.2,(((Felis_margarita:5.3,Felis_silvestris:5.3):0.9,Felis_chaus:6.2):2.6,Caracal_caracal:8.8):2.3):3.8,Puma_concolor:14.9):23.7,Crocuta_crocuta:38.6):3,((Suricata_suricatta:35.7,Galidia_elegans:35.7):1.1,((Genetta_genetta:23.9,Paradoxurus_hermaphroditus:23.9):12.7,Cryptoprocta_ferox:36.6):0.2):4.8):21.8):18,Manis_pentadactyla:81.4):3.2):0.2,((Desmodus_rotundus:31,Carollia_perspicillata:31):29,(Tadarida_brasiliensis:54,(Miniopterus_schreibersi:51.6,(Nycticeius_humeralis:26,Antrozous_pallidus:26,(Nyctalus_lasiopterus:5.5,Nyctalus_noctula:5.5):20.5,Pipistrellus_pipistrellus:26,(Eptesicus_fuscus:9.5,Eptesicus_serotinus:9.5):16.5,(Myotis_grisescens:10.9,Myotis_lucifugus:10.9,Myotis_velifer:10.9,(Myotis_myotis:8,Myotis_thysanodes:8):2.9):15.1):25.6):2.4):6):24.8):3,(Atelerix_albiventris:75.4,(((Crocidura_fuscomurina:12.3,Crocidura_russula:12.3,Crocidura_suaveolens:12.3):6.1,(Suncus_etruscus:11.5,Suncus_murinus:11.5):6.9):28.2,((Sorex_cinereus:13.7,Sorex_vagrans:13.7):21.2,(Blarina_brevicauda:23.6,Cryptotis_parva:23.6):11.3):11.7):28.8):12.4):8.3):2.2,(Choloepus_didactylus:70.5,Myrmecophaga_tridactyla:70.5,(Chaetophractus_villosus:32.3,Tolypeutes_matacus:32.3):38.2):27.8):0.2,(((((Microgale_dobsoni:14.7,Microgale_talazaci:14.7):42.9,(Tenrec_ecaudatus:31.4,(Setifer_setosus:14.8,Echinops_telfairi:14.8):16.6):26.2):30.7,Elephantulus_rufescens:88.3):1.9,Orycteropus_afer:90.2):0.2,((Trichechus_manatus:74.1,(Dendrohyrax_dorsalis:18.6,Procavia_capensis:18.6):55.5):1.7,Loxodonta_africana:75.8):14.6):8.1):10;

References

Bininda-Emonds ORP, Cardillo M, Jones KE, MacPhee RDE, Beck RMD, et al. (2007) The delayed rise of present-day mammals. Nature 446: 507-512.

Bininda-Emonds ORP, Cardillo M, Jones KE, MacPhee RDE, Beck RMD, et al. (2008) The delayed rise of present-day mammals (vol 446, pg 507, 2007). Nature 456: 274-274.

**Phylogenetic information used in PGLS regression analyses for Marsupials (Newick format):**

(((((((Acrobates_pygmaeus:32.6,(Petaurus_breviceps:5.3,Petaurus_norfolcensis:5.3):27.3):12.5,Trichosurus_vulpecula:45.1):0.7,(Bettongia_lesueur:24.8,((((Macropus_parma:9,((Macropus_rufus:7.2,Macropus_robustus:7.2):1.3,(Macropus_fuliginosus:2.7,Macropus_giganteus:2.7):5.8):0.5):0.2,Wallabia_bicolor:9.2):0.2,Setonix_brachyurus:9.4):0.3,Thylogale_billardierii:9.7):15.1,Potorous_tridactylus:24.8):21):8.3,Phascolarctos_cinereus:54.1):9.6,Antechinus_flavipes:63.7):2.1,((Isoodon_obesulus:7.4,Isoodon_macrourus:7.4):5,(Perameles_gunnii:7.7,Perameles_nasuta:7.7):4.7):53.4):16.7,Didelphis_virginiana:82.5):10;

References

Bininda-Emonds ORP, Cardillo M, Jones KE, MacPhee RDE, Beck RMD, et al. (2007) The delayed rise of present-day mammals. Nature 446: 507-512.

Bininda-Emonds ORP, Cardillo M, Jones KE, MacPhee RDE, Beck RMD, et al. (2008) The delayed rise of present-day mammals (vol 446, pg 507, 2007). Nature 456: 274-274.

**Phylogenetic information used in PGLS regression analyses for reptiles (Newick format):**

(Sphenodon_punctatus:228,((Xantusia_vigilis:151.807985,(Plestiodon_fasciatus:87.343386,(Oligosoma_zelandicum:44.344626,Emoia_atrocostata:44.344625):42.998761):64.4646):14.80675,((Aspidoscelis_tigris:86.265567,Gymnophthalmus_speciosus:86.265569):75.946911,((((Varanus_griseus:29.895161,Varanus_niloticus:29.89516):2.281204,(Varanus_salvator:26.178837,((Varanus_komodoensis:12.925442,Varanus_varius:12.925442):9.454622,Varanus_brevicauda:22.380065):3.798772):5.997527):124.984149,((Dipsosaurus_dorsalis:66.283708,(Iguana_iguana:29.908167,Sauromalus_ater:29.908168):36.375542):33.20995,(((Callisaurus_draconoides:38.666362,Phrynosoma_solare:38.666361):16.056385,(Uta_stansburiana:46.042313,(Sceloporus_graciosus:24.379867,(Sceloporus_magister:21.197453,Sceloporus_undulatus:21.197454):3.182415):21.662444):8.680433):38.213185,Anolis_acutus:92.935932):6.557728):57.666854):3.41293,((Boa_constrictor:78.123018,((Python_molurus:16.621514,Python_sebae:16.621514):12.418983,Broghammerus_reticulatus:29.040497):49.08252):6.025844,(Crotalus_viridis:61.540342,((Spalerosophis_diadema:37.00154,(Coluber_constrictor:35.748009,Bogertophis_subocularis:35.74801):1.253533):14.412247,((Nerodia_erythrogaster:9.824183,Thamnophis_sirtalis:9.824183):40.377705,(Diadophis_punctatus:39.854591,Heterodon_platirhinos:39.854591):10.347298):1.2119):10.126553):22.608519):76.424582):1.639035):4.402257):61.385264);

References

Pyron RA, Burbrink FT (2013) Early origin of viviparity and multiple reversions to oviparity in squamate reptiles. Ecol Lett doi 10.1111/ele.12168.

**Phylogenetic information used in PGLS regression analyses for fishes (Newick format):**

(Acipenser_fulvescens:0.1694224042,(((Esox_lucius:0.1652276387,(Coregonus_clupeaformis:0.02207660207,Salvelinus_alpinus:0.03339327082):0.04601017738):0.1746225514,Mallotus_villosus:0.2641026346):0.02038966079,((Myctophum_punctatum:0.06188001979,Benthosema_glaciale:0.05720280118):0.2540195337,((Merluccius_productus:0.0956247661,(Pollachius_virens:0.01792862433,(Gadus_morhua:0.01519847533,Melanogrammus_aeglefinus:0.0186296665):0.003823482864):0.1271970856):0.2594421838,((Pseudupeneus_maculatus:0.298528628,(Scomber_japonicus:0.07980301979,(Thunnus_albacares:0.01448124136,Katsuwonus_pelamis:0.02139920043):0.0426133664):0.03279937595):0.01619328388,(((Solea_solea:0.1416758351,(Eopsetta_jordani:0.02155630744,(Pseudopleuronectes_americanus:0.01862468133,Pleuronectes_platessa:0.01934174353):0.01759611408):0.09948556298):0.07957520024,(Cololabis_saira:0.234600509,Mugil_cephalus:0.1632975407):0.02823690333):0.006496572546,((Dicentrarchus_labrax:0.08153384991,(Balistes_vetula:0.2265597188,(Haemulon_plumierii:0.0845117254,(Apsilus_dentatus:0.06088102846,Ocyurus_chrysurus:0.0640180576):0.02266789818):0.02521235057):0.002980752426):0.006906368512,(Cephalopholis_fulva:0.09737822789,(Perca_fluviatilis:0.1604762411,(Pungitius_pungitius:0.04495485821,Gasterosteus_aculeatus:0.04412082057):0.1635325289):0.003469841719):0.01996538976):0.02035081766):0.009813312277):0.150946498):0.01845526339):0.1286618662):0.5398552204);

References

Betancur-R. R BR, Wiley EO, Carpenter K, López JA, Li C, Holcroft NI, Arcila D, Sanciangco M, Cureton II JC, Zhang F, Buser T, Campbell MA, Ballesteros JA, Roa-Varon A, Willis S, Borden WC, Rowley T, Reneau PC, Hough DJ, Lu G, Grande T, Arratia G, Ortí G (2013) The tree of life and a new classification of bony fishes. PLoS Currents: Tree of Life doi:10.1371/currents.tol.53ba26640df0ccaee75bb165c8c26288.

**Phylogenetic information used in PGLS regression analyses for non-avian dinosaurs (Newick format):**

(((((((AlamosaurusA:0,(AlamosaurusC:0,AlamosaurusB:0):0):90.2,Camarasaurus:43.67):5.5,((DiplodicideaA:0,DiplodicideaB:0):71.9,(ApatosaurusB:0,ApatosaurusA:0):15.7):0):0,Mamenchisaurus:52.2):68.8,((PlateosaurusA:0,PlateosaurusB:0):19.9,Massospondylus_carinatus:31.9):8.5):5,(((Gorgosaurus_libratus:18,Albertosaurus_sacrophagus:18):0,Tyrannosaurus_rex:18):67.3,(ArchaeopteryxB:0,ArchaeopteryxA:0):25.4):84.2):2.5,(Psittacosaurus_luijatunensis:2.99,Psittacosaurus_mongoliensis:13.42):112.05);

Note: Dinosaur species marked with a letter correspond to the dinosaur species marked with the same letter in Table S1.

References

Topology was constructed from Lloyd GT, Davis KE, Pisani D, Tarver JE, Ruta M, et al. (2008) Dinosaurs and the Cretaceous Terrestrial Revolution. Proc Roy Soc B-Biol Sci 275: 2483-2490.

Age ranges (branch lengths) were taken from Paleobiology_Database (2013). http://paleodb.org. Accessed 09.09.2013-12.09.2013.:

Plateosaurus 215.56 to 201.6 Ma http://paleodb.org/?a=checkTaxonInfo&taxon_no=38644&is_real_user=1 09.09.2013

Plateosaurus engelhardti 221.500 to 205.600 Ma http://paleodb.org/bridge.pl?a=checkTaxonInfo&taxon_no=56683&is_real_user=1

Mamenchisaurus 161.2 to 109.0 Ma http://paleodb.org/?a=checkTaxonInfo&taxon_no=38670&is_real_user=1 09.09.2013

Apatosaurus 155.700 to 145.500 Ma http://paleodb.org/bridge.pl?a=checkTaxonInfo&taxon_no=38665&is_real_user=1

Camarasaurus 155.700 to 112.030 Ma http://paleodb.org/bridge.pl?a=checkTaxonInfo&taxon_no=38697&is_real_user=1

Alamosaurus 83.500 to 65.500 Ma http://paleodb.org/bridge.pl?a=checkTaxonInfo&taxon_no=38683&is_real_user=1

Diplodocidae (diplodocid) 161.200 to 89.300 Ma http://paleodb.org/bridge.pl?a=checkTaxonInfo&taxon_no=38664&is_real_user=1 12.09.2013

Archeopteryx 150.800 to 125.450 Ma http://paleodb.org/bridge.pl?a=checkTaxonInfo&taxon_no=39240&is_real_user=1

Psittacosaurus luijatunensis 125.450 to 122.460 Ma http://paleodb.org/bridge.pl?a=checkTaxonInfo&taxon_no=109454&is_real_user=1

Psittacosaurus mongoliensis 125.450 to 112.030 Ma http://paleodb.org/bridge.pl?a=checkTaxonInfo&taxon_no=52831&is_real_user=1

Massospondylus carinatus 196.500 to 189.600 Ma http://paleodb.org/bridge.pl?a=checkTaxonInfo&taxon_no=56595&is_real_user=1

Albertosaurus sacrophagus 83.500 to 65.500 Ma http://paleodb.org/bridge.pl?a=checkTaxonInfo&taxon_no=38607&is_real_user=1

Gorgosaurus libratus 83.500 to 65.500 Ma http://paleodb.org/bridge.pl?a=checkTaxonInfo&taxon_no=53194&is_real_user=1

Tyrannosaurus rex 83.500 to 65.500 Ma http://paleodb.org/bridge.pl?a=checkTaxonInfo&taxon_no=54833&is_real_user=1

Theropoda 247.2 http://paleodb.org/bridge.pl?a=checkTaxonInfo&taxon_no=91970&is_real_user=1

sauropodomorpha 242.000 to 33.900 Ma http://paleodb.org/bridge.pl?a=checkTaxonInfo&taxon_no=91972&is_real_user=1

prosauropoda 247.200 to 33.900 http://paleodb.org/bridge.pl?a=checkTaxonInfo&taxon_no=162292&is_real_user=1

Sauropoda 221.500 to 48.600 Ma http://paleodb.org/bridge.pl?a=checkTaxonInfo&taxon_no=95571&is_real_user=1

Ornithischia 235.000 to 65.500 Ma http://paleodb.org/bridge.pl?a=checkTaxonInfo&taxon_no=91973&is_real_user=1

Saurischia 247.200 to 0.000 Ma http://paleodb.org/bridge.pl?a=checkTaxonInfo&taxon_no=91969&is_real_user=1
